# Controlled Carbon Dioxide Terpolymerizations to Deliver Toughened yet Recyclable Thermoplastics

**DOI:** 10.1021/acs.macromol.4c00455

**Published:** 2024-04-24

**Authors:** Kam C. Poon, Madeleine L. Smith, Charlotte K. Williams

**Affiliations:** Chemistry Research Laboratory, Department of Chemistry, University of Oxford, Oxford OX1 3TA, United Kingdom

## Abstract

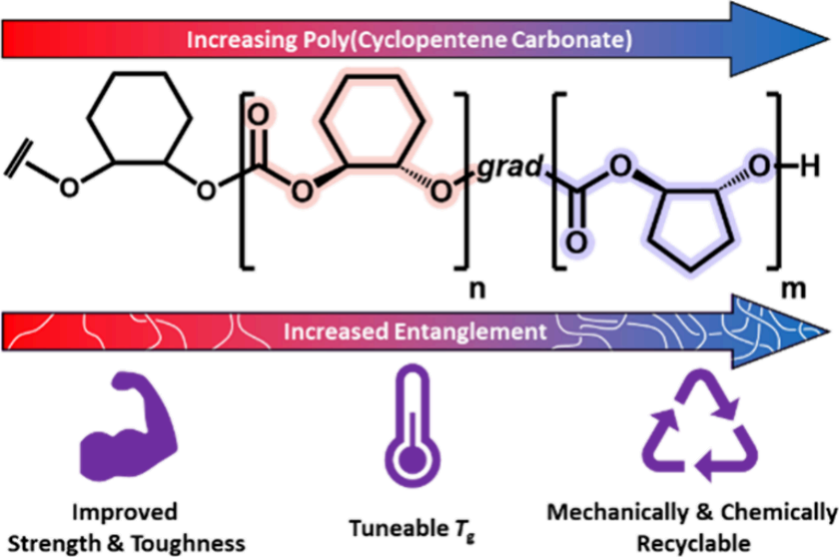

Using CO_2_ polycarbonates as engineering thermoplastics has been limited by their mechanical performances, particularly their brittleness. Poly(cyclohexene carbonate) (PCHC) has a high tensile strength (40 MPa) but is very brittle (elongation at break <3%), which limits both its processing and applications. Here, well-defined, high molar mass CO_2_ terpolymers are prepared from cyclohexene oxide (CHO), cyclopentene oxide (CPO), and CO_2_ by using a Zn(II)Mg(II) catalyst. In the catalysis, CHO and CPO show reactivity ratios of 1.53 and 0.08 with CO_2_, respectively; as such, the terpolymers have gradient structures. The poly(cyclohexene carbonate)-*grad*-poly(cyclopentene carbonate) (PCHC-*grad*-PCPC) have high molar masses (86 < *M*_n_ < 164 kg mol^–1^, *Đ*_M_ < 1.22) and good thermal stability (*T*_d_ > 250 °C). All the polymers are amorphous with a single, high glass transition temperature (96 < *T*_g_ < 108 °C). The polymer entanglement molar masses, determined using dynamic mechanical analyses, range from 4 < *M*_e_ < 23 kg mol^–1^ depending on the polymer composition (PCHC:PCPC). These polymers show superior mechanical performance to PCHC; specifically the lead material (PCHC_0.28_-*grad*-PCPC_0.72_) shows 25% greater tensile strength and 160% higher tensile toughness. These new plastics are recycled, using cycles of reprocessing by compression molding (150 °C, 1.2 ton m^–2^, 60 min), four times without any loss in mechanical properties. They are also efficiently chemically recycled to selectively yield the two epoxide monomers, CHO and CPO, as well as carbon dioxide, with high activity (TOF = 270–1653 h^–1^, 140 °C, 120 min). The isolated recycled monomers are repolymerized to form thermoplastic showing the same material properties. The findings highlight the benefits of the terpolymer strategy to deliver thermoplastics combining the beneficial low entanglement molar mass, high glass transition temperatures, and tensile strengths; PCHC properties are significantly improved by incorporating small quantities (23 mol %) of cyclopentene carbonate linkages. The general strategy of designing terpolymers to include chain segments of low entanglement molar mass may help to toughen other brittle and renewably sourced plastics.

## Introduction

The development of new sustainable polymeric materials to replace the environmentally persistent petroleum-derived polymers, ubiquitous in our lives, is an ever-urgent global challenge.^1−3^ Such renewably sourced polymers are key to helping reduce the 1.8 Gt of CO_2_ equivalents emitted annually from polymer production worldwide.^4,5^ One attractive class is polymers produced directly from carbon dioxide (CO_2_) by the catalyzed ring opening copolymerization (ROCOP) of CO_2_ and heterocycles.^6−10^ CO_2_ is a cheap and abundant waste feedstock suitable for use in existing manufacturing infrastructure.^11^ When used in the ROCOP with epoxides, CO_2_ allows for a threefold reduction in greenhouse gas emissions relative to the corresponding polyether: for every molecule of CO_2_ polymerized, two more are saved through the replacement of epoxide.^12−14^ Furthermore, recent reports have shown that these materials can be chemically recycled to monomer, helping to facilitate a future circular economy for plastics.^15,16^

To date, CO_2_-derived polymers are almost exclusively explored for uses as low molar mass (*M*_n_) polyols, for example in the production of polyurethanes as foams, elastomers, adhesives, and coatings.^17−23^ While specific higher molar mass CO_2_-derived polycarbonates, such as PCHC, are available commercially, they are employed as sacrificial binders. PCHC could be a useful engineering plastic since it has a high glass transition temperature (*T*_g_ = 126 °C), high tensile strength (40 MPa), and high Young’s modulus (2.2 GPa). However, it is very brittle with a very low elongation at break (<3%) and this limits its effective processing and use.^24,25^ If these materials are to find uses in other applications, their mechanical properties must be better understood, improved, and expanded.

Only a few high molar mass CO_2_-derived polycarbonates have been reported, and even in those cases, the polymers’ mechanical properties were usually not investigated. In 2019, Feng and co-workers synthesized “ultrahigh” molar mass PCHC (*M*_n_ ∼450 kg mol^–1^, *Đ*_M_ = 1.31) through rigorous drying of both CO_2_ and CHO, using triisobutylaluminum and triethylborane, respectively.^26^ Li and co-workers also produced high molar mass PCHC (*M*_n_ ∼ 280 kg mol^–1^, *Đ*_M_ = 1.59), in a similar manner in 2022.^27^ Rieger et al. and Greiner et al. both reported high molar mass (*M*_n_ > 100 kg mol^–1^), bioderived poly(limonene) carbonate, and terpolymers with PCHC.^28−31^ While these reports are synthetically impressive, they do not address high molar mass PCHC mechanical characterization.

In 2001, Darensbourg et al. reported PCHC with moderate molar mass and high dispersity (*M*_n_ = 42 kg mol^–1^, *Đ*_M_ = 6), which exhibited an ultimate tensile strength of 43 MPa, a Young’s modulus of 3.3 GPa, and a strain at break of 2%.^24^ The authors predicted the molar mass between entanglements (*M*_e_) to be 16 kg mol^–1^ but noted that the value was likely to be an underestimate. In 2022, Frey et al. reported on PCHC chain dynamics through experimental and computational investigations, highlighting that these were dominated by the cyclohexene ring conformational changes.^25^ The authors synthesized PCHC with intermediate molar mass (5 < *M*_n_ < 33 kg mol^–1^) and predicted that *M*_e_ should be far above 16 kg mol^–1^, noting that materials that have higher molar masses are needed to more accurately determine a value of *M*_e_. We recently reported the synthesis of high-*M*_n_, narrowly dispersed CO_2_-derived PCHC and poly(cyclopentene carbonate) PCPC, using a Co(II)Mg(II) catalyst that has high activity and selectivity.^32^ The PCHC and PCPC samples, both having *M*_n_ ∼100 kg mol^–1^, were investigated using oscillatory rheology to estimate the *M*_e_ (from time–temperature superposition (TTS) master curves, eq S1). The results were striking, with an order of magnitude difference in *M*_e_ values between PCHC, where *M*_e_ = 56 kg mol^–1^, and PCPC, where *M*_e_ = 4.0–4.9 kg mol^–1^. As such, in that study, only the PCPC sample was expected to be above the critical molar mass (*M*_c_).

PCPC remains a far less explored polymer than PCHC, with a few reports of its use in catalysis. For example, Wu and co-workers synthesized PCPC with *M*_n_ up to 84 kg mol^–1^, and Lu and co-workers have reported the synthesis of semicrystalline, stereocomplex PCPC, with a high melting temperature of 199 °C.^33,34^ Despite these reports, the potential of PCPC as a tough engineering plastic is yet to be fully realized. Comparing PCPC to PCHC reveals that it has a slightly higher CO_2_ content (34 vs 31 wt %, respectively), a significantly lower zero shear viscosity (0.8 vs 90 MPa s, respectively), a higher ultimate tensile strength (59 vs 40 MPa, respectively), and a slightly higher strain at break (7 and 3%, respectively), resulting in a greater tensile toughness (2.9 and 0.9 MJ m^–3^, respectively).^32,34^ In fact, only the Young’s Modulus and *T*_g_ of PCPC are lower than those of PCHC (1.7 vs 2.2 GPa, 85 vs 126 °C respectively). The PCPC properties motivated the current study, where terpolymerizations incorporating CHO, CPO, and carbon dioxide target higher molar mass polycarbonates. The objective was to understand how terpolymer compositions and structures might influence chain entanglement and, subsequently, thermomechanical properties with the goal to increase toughness.

Recently, the catalyzed chemical recycling of high molar mass PCHC, and PCPC, to form epoxides and CO_2_ was reported.^32^ This route allows for the synthesis of recycled polymers showing properties equivalent to those of the virgin materials. For PCPC, Darensbourg and co-workers reported the first depolymerization catalyst forming CPO and CO_2_.^35,36^ Subsequently, other depolymerization catalysts were reported for both PCHC and PCPC.^15,16,33,34,37−39^ We reported a very selective solid-state depolymerization of high molar mass PCHC and PCPC, using a high activity Mg(II)Co(II) catalyst.^32^ Here, the target terpolymers should be investigated for both mechanical and chemical recycling.

## Results

**Figure 1:**
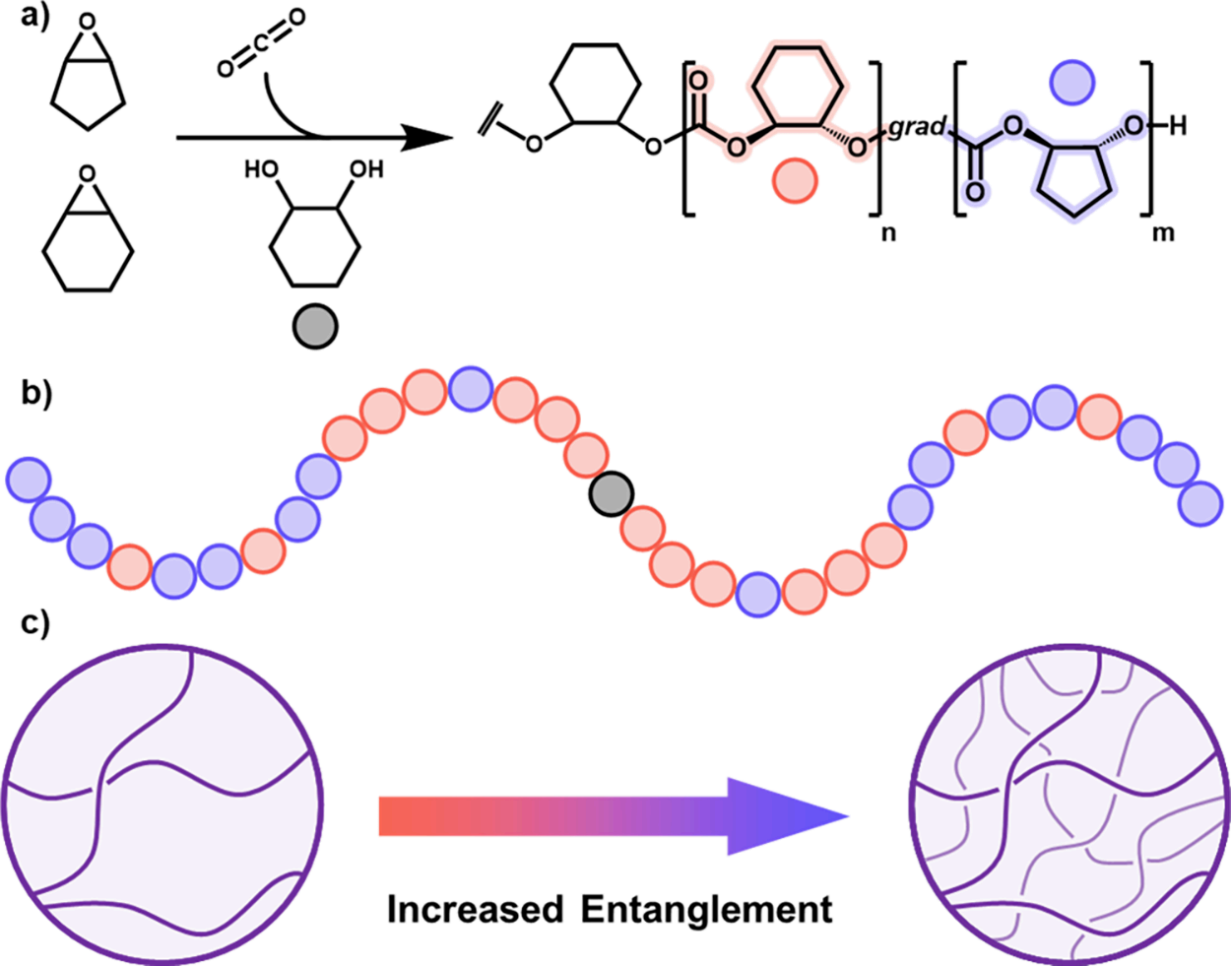
(a) Synthesis of PCHC-*grad*-PCPC terpolymers. ROCOP of CO_2_, CHO, and CPO. Polymerization conditions: [LZnMg(C_6_F_5_)_2_] catalyst, with cyclohexene diol (CHD) as initiator, where [Cat]_0_:[CHD]_0_:[CHO + CPO]_0_ = 1:4:10,000, [CHO + CPO]_0_ = 4 M, toluene, 80 °C. Schematic representation of (b) PCHC-*grad*-PCPC terpolymer and (c) increasingly entangled networks.

To make the terpolymers from CHO, CPO, and CO_2_, we selected the previously reported [LZnMg(C_6_F_5_)_2_] catalyst due to its high activity, selectivity, ease of synthesis, end-group selectivity, and ability to produce polycarbonates that have a high molar mass, are monomodal, and have low *Đ*_M_.^40^ The catalyst features aryl coligands that cannot initiate polymerization; rather, they react with an added diol, 1,2-cyclohexane diol (CHD), to form the true initiator *in situ*. This strategy results in the highest levels of initiation control, essential to deliver monodisperse terpolymer samples that have a high molar mass. To make the terpolymers, the catalyst [LZnMg(C_6_F_5_)_2_], cyclohexene diol, and both monomers CHO and CPO were dissolved in toluene ([cat.]_0_ = 0.3 mM, [CHO + CPO]_0_ = 4 M, [cat.]:[CHD]:[CHO + CPO] = 1:4:10,000). The polymerizations were conducted in a steel Parr reactor, at 80 °C and under a CO_2_ atmosphere (40 bar) for 96 h; conditions were selected to maximize productivity (conversion) (Figure 1). Since these are controlled polymerizations, varying the starting monomer feed ratios, i.e., CHO:CPO, should result in terpolymers with predictable PCHC:PCPC (Table S1).

**Figure 2:**
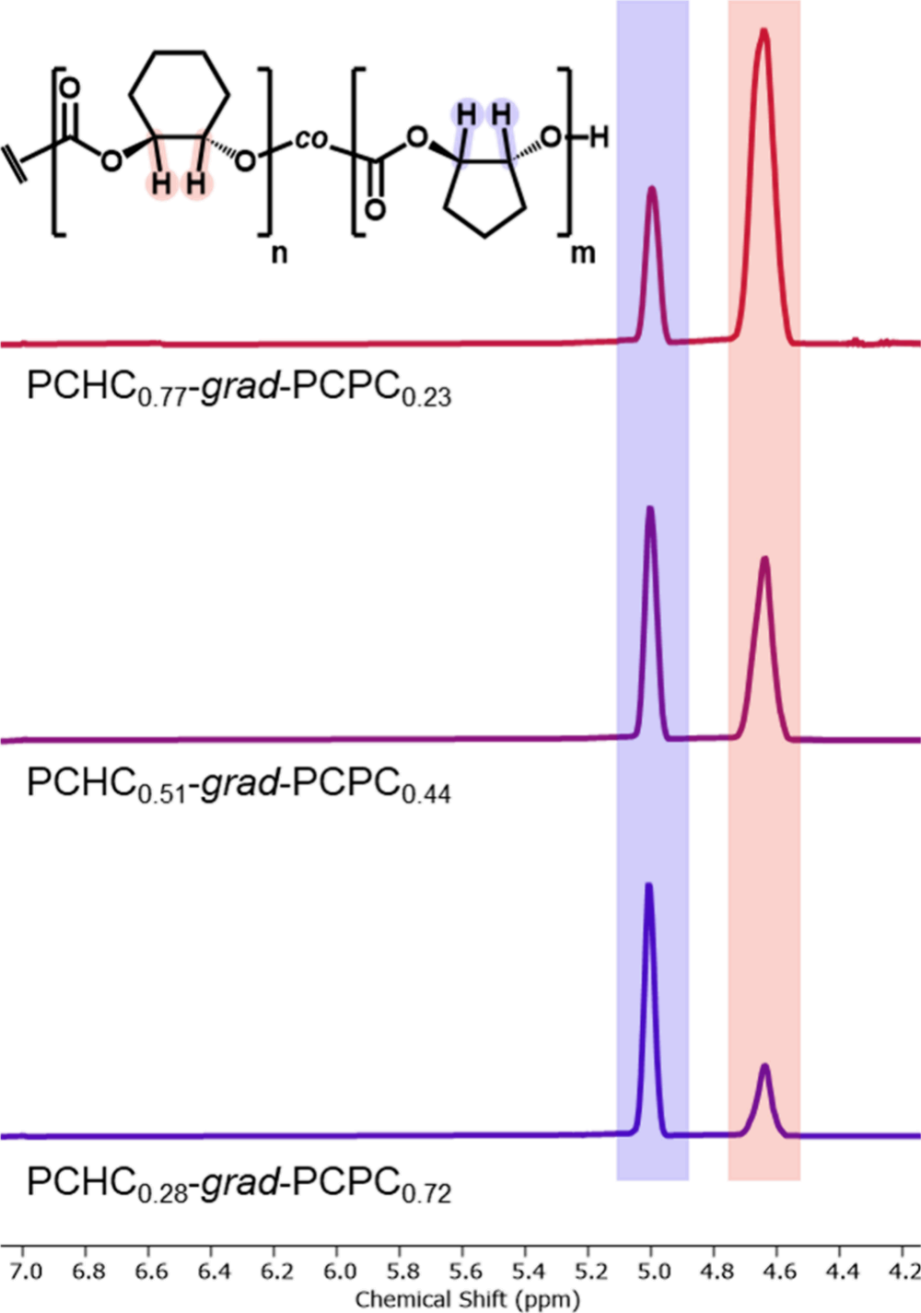
^1^H NMR (400 MHz, CDCl_3_) spectra of PCHC-*grad*-PCPC terpolymers with the purple highlighting the PCHC and pink the PCPC resonances used to determine terpolymer composition (for full spectra, see Figures S1–S4).

The polymerizations were stopped by reducing the temperature and depressurizing the reactors; the crude products were analyzed by ^1^H NMR spectroscopy. For all materials synthesized, >95% epoxide conversion was achieved. The materials were then isolated by precipitation in MeOH. The ^1^H NMR spectrum of the purified materials established the PCHC and PCPC contents in each of the materials, by comparing the integrals of the methine proton signals at δ 4.65 (PCHC) and 5.00 (PCPC), respectively (Figure 2). The PCHC:PCPC values are 77:23, 51:49, and 28:72. The PCHC–PCPC terpolymers were also fully characterized by ^1^H NMR and ^13^C NMR spectroscopy (Figures S1–S4). The absence of resonances corresponding to polyether (3.45 ppm) or cyclic carbonate (4.00 and 4.68 ppm) indicated there were >99% CO_2_ selectivity and >99% polymer selectivity. Size exclusion chromatography (SEC) confirmed the formation of samples showing high molar masses, with *M*_n_ > 85 kg mol^–1^, monomodal distributions with narrow dispersity (*Đ*_M_ < 1.22) (Table 1B, Figure S5). The terpolymer structure was also indicated by ^1^H DOSY NMR spectroscopy with a single diffusion coefficient observed for PCHC and PCPC signals (Figure S6).

**Table 1:** Polycarbonate Material Characterization Data

polymer	a*M*_n_^b^[*Đ*_M_] (kg mol^–1^)	c*T*_g,DSC_ (° C)	d*T*_d,5%_ (° C)	e*E*_Y_ (GPa)	fσ (MPa)	gε_b_ (%)	h*U*_T_ (MJ m^–3^)	i*M*_e_ (kg mol^–1^)
PCHC	121.6 [1.09]	126	265	2.16 ± 0.06	40.0 ± 1.8	3.3 ± 0.3	0.9 ± 0.1	27.6 ± 4.4
PCHC_0.77_-*grad*-PCPC_0.23_	164.2 [1.21]	108	274	1.48 ± 0.06	50.1 ± 3.2	6.8 ± 1.3	2.36 ± 0.5	22.7 ± 3.6
PCHC_0.51_-*grad*-PCPC_0.49_	103.8 [1.12]	105	250	1.55 ± 0.15	49.4 ± 3.4	6.5 ± 0.9	2.34 ± 0.6	10.7 ± 1.7
PCHC_0.28_-*grad*-PCPC_0.72_	086.9 [1.05]	96	255	1.70 ± 0.24	52.0 ± 3.7	7.4 ± 1.3	2.81 ± 0.6	4.3 ± 0.7
PCPC	114.4 [1.06]	85	271	1.7 ± 0.09	58.5 ± 1.7	7.1 ± 1.9	2.9 ± 1.1	3.2 ± 0.5

a Determined from SEC analysis (THF); calibrated with narrow polystyrene standards (Figure S5).

b *M*_w_/*M*_n_. Higher *M*_n_ is a result of higher epoxide conversion and lower residual diol content. All terpolymers above critical molar mass (*M*_c_).

c Glass transition temperature determined from the midpoint of the second DSC heating cycle.

d Thermal degradation is the temperature at 5% mass loss, determined by TGA. Specimens suitable for uniaxial tensile testing were solvent cast, dried, and compression molded (150 °C, 1.2 tons m^–2^, 60 min, Supporting Information). Measurements conducted independently on 10 specimens.

e Young’s modulus.

f Tensile strength.

g Strain at break.

h Tensile toughness (area under the stress–strain curve). Mean values ± std. dev.

i Entanglement molecular weight determined by DMA temperature ramps; ranges in values reflect the assumed melt density range of 800–1100 kg m^–3^ (eq S1, Figures S10–S14, and Table S3).

**Figure 3:**
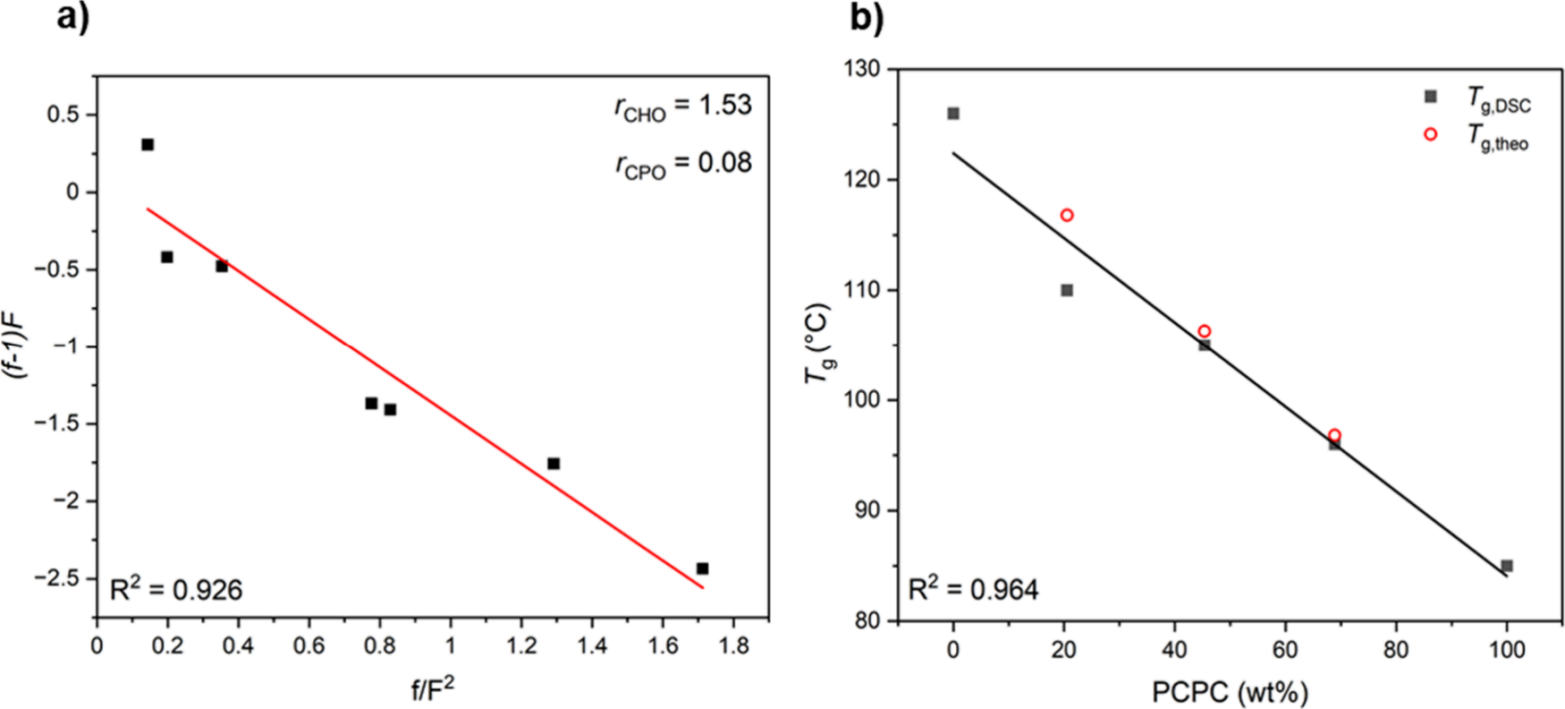
(a) Fineman–Ross plots for CHO, CPO, and CO_2_ terpolymerizations at 80 °C, where the gradient equals −*r*_CHO_ and the intercept equals *r*_CPO_. (b) Plot of the *T*_g_ values for PCHC-*grad*-PCPC, from DSC, vs the PCPC content. The experimental data (black squares) are compared with *T*_g,theo_ values (red circles) determined using the Fox–Flory relationships.

To better understand the terpolymer structures, Fineman–Ross kinetic analysis was used to determine the epoxides’ reactivity ratios in the ROCOP.^41,42^ Terpolymerizations were conducted using the same catalyst and initiator at 1 bar CO_2_ pressure, with varying starting quantities of CHO and CPO. By comparing the CHO:CPO feed ratios (*F*) to the ratio of the monomers enchained in the terpolymer (*f*) at low conversion (<15% total epoxide consumption), the Fineman–Ross plot was constructed (Figure 3a, Table S2, and eq S2). The relative ratios, *F* and *f*, were determined from the ^1^H NMR spectra of aliquots taken from the terpolymerizations, at *t* = 0 and 120 min, respectively. Subsequently, the reactivity ratios, *r*_CHO_ and *r*_CPO_,were determined as 1.53 and 0.08 for the Zn(II)Mg(II) catalyst, respectively.^43^ As *r*_CHO_ > 1 > *r*_CPO_, compositional drift should result in a gradient polycarbonate structure. In the initial stages of the copolymerization, CHO is incorporated faster than CPO; however, as CHO is depleted, increasing quantities of CPO are enchained in the terpolymer. Using a ^31^P{^1^H} NMR titration method, the chain-end groups were analysed and they all correspond to PCPC–OH, which is fully consistent with the proposed gradient terpolymer structure (Figure S7). Henceforth, terpolymers are abbreviated as PCHC_0.*x*_-grad-PCPC_0._*_y_,* where *x* and *y* are the molar ratios of the two carbonates.

The glass transition temperatures (*T*_g_) of the gradient terpolymers were determined by differential scanning calorimetry (DSC) (Table 1). All materials are amorphous and a single *T*_g_ was observed, its value gradually decreases as the PCPC content increases, falling from 110 °C for PCHC_0.77_-*grad*-PCPC_0.23_ to 96 °C for PCHC_0.28_-*grad*-PCPC_0.72_ (Figure 3b and Figure S8). The experimental *T*_g_ values are fully consistent with the theoretical values (*T*_g,theo_) predicted by the Flory–Fox equation for each terpolymer (eq S3). As such, the PCHC and PCPC segments are expected to be miscible in the terpolymers. This segmental miscibility is proposed to be critical to controlling the chain entanglement, *M*_e_, since it allows for interchain PCHC and PCPC interactions. All the terpolymers exhibited good thermal stability, with all *T*_d,5%_ values exceeding 250 °C (Figure S9). Therefore, the material processing temperature range is >143 °C for all the terpolymers.

**Figure 4:**
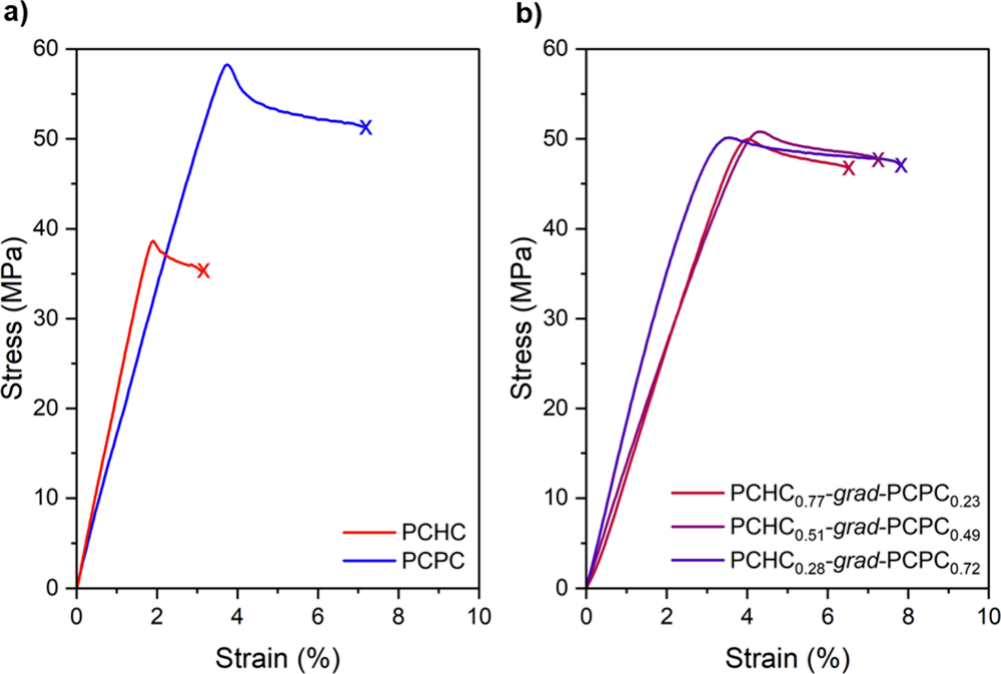
Representative stress–strain curves (10 mm min^–1^) for (a) PCHC and PCPC homopolymers and (b) PCHC-*grad*-PCPC terpolymers.

To probe the influences of the terpolymer composition on the tensile mechanical properties of the materials, specimens were subjected to uniaxial extension experiments, according to ISO 527 (10 mm min^–1^) (Table 1 and Figure 4). Thin terpolymer films were solvent cast from THF (20 wt % solution) and dried under vacuum to remove all solvent residues (120 °C, 48 h). ^1^H NMR spectroscopy confirmed the absence of residual THF. The terpolymers were subsequently compression molded (150 °C, 1.2 tons, 60 min) to produce samples suitable for mechanical testing. The PCHC_0.77_-*grad*-PCPC_0.23_, PCHC_0.51_-*grad*-PCPC_0.49_, and PCHC_0.28_-*grad*-PCPC_0.72_ all exhibit high Young’s moduli (∼1.6 GPa), ultimate tensile strengths (∼50 MPa), elongation at break values (∼7%), and tensile toughness values (∼2.5 MJ m^–3^) that are within error of each other, despite the varying polycarbonate compositions. All of the gradient terpolymers display tensile strengths halfway between those for high *M*_e_ PCHC (∼40 MPa) and low *M*_e_ PCPC (∼60 MPa). Critically, even at the lowest levels of PCPC content, the strain at break for the gradient terpolymers is significantly greater than that of PCHC, and, within error, of the 7.1% for PCPC. As a result, the tensile toughness of all these terpolymers is >200% greater than that of PCHC. This improvement in toughness for PCHC, even with the introduction of low quantities of PCPC linkages (23 mol %), results in minimal compromise to the terpolymer glass transition temperatures and, crucially, is easily implemented using the catalyzed polymerization.

**Figure 5:**
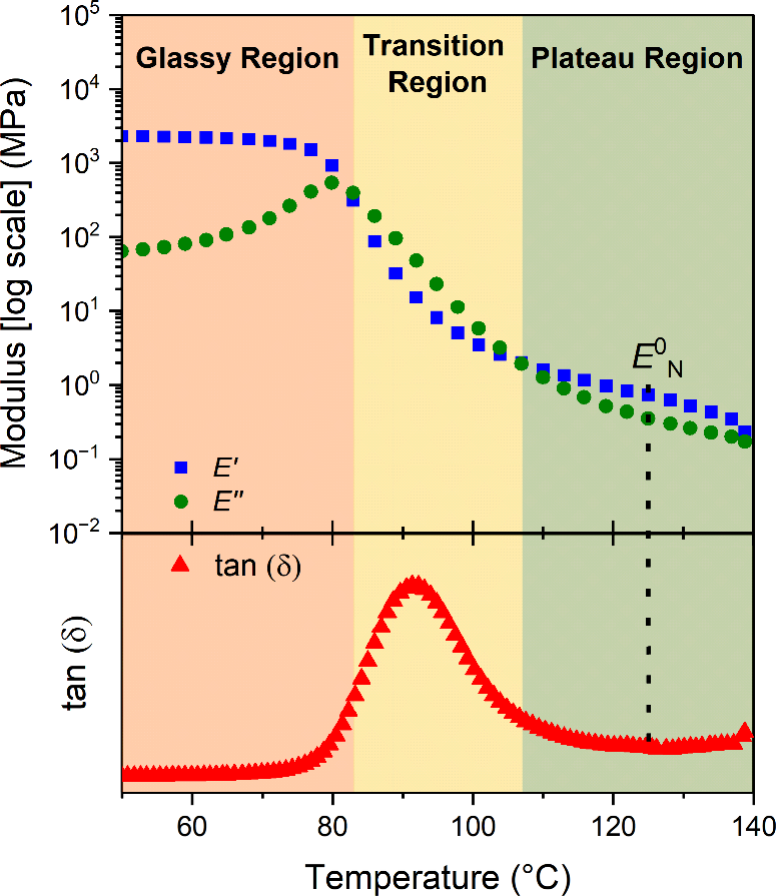
Example DMA temperature ramp profile for PCHC_0.51_-*grad-*PCPC_0.49_. Glassy, transition, and plateau regions are highlighted in orange, yellow, and green, respectively. *E*^0^_N_ is extracted from the rubbery plateau at a minimum of tan(δ).

To better understand these trends in mechanical performance, all polycarbonates were subjected to dynamic mechanical analysis (DMA) temperature ramps. Thin films were cut using two parallel blades, placed in tension clamps, and heated from −80 to 250 °C (or until the material deformed beyond the limits of the geometry), at a rate of 3 °C min^–1^, with a frequency of 1 Hz, 0.1 N preload force, and 0.1% strain amplitude (Figure 5 and Figures S10–S14). Below the glass transition of each polycarbonate, the storage modulus remains above the loss modulus, with both values reaching a plateau despite the increasing temperature. In this region, the terpolymers are glassy and chain movement is severely restricted. As the material passes through its glass transition, the storage modulus decreases, while the loss modulus increases (resulting in a peak in the tan (δ)). In this transition region, terpolymer chains start to move with short-range rearrangements dominating and the material transitions from a glassy to rubbery state. Beyond this region, the storage modulus is once again greater than the loss modulus. This is the plateau region where the polymer network is bound by molecular entanglements between the polymer chains. As temperatures increase further, there is sufficient energy for polymer chains to move through any topological restrictions and the network loses mechanical integrity, i.e., the terminal region. By extracting the value of storage modulus at the minimum of tan(δ) in the plateau region, *E*^0^_N_ was determined. This value was converted to the sheer modulus using eq S4, where ν is the Poisson’s ratio of the material, estimated as 0.36, for all polycarbonates.^44^ As such, a value of *M*_e_ was estimated using eq S1 (Table S3).

It is important to note that *M*_e_ is often reported from TTS master curves constructed from numerous rheological frequency sweeps at various temperatures. Deducing *M*_e_ by DMA is often more challenging, as high molecular weight materials are required to ensure that the plateau region is observed before the material deforms. Here, the high molecular weights of these terpolymers obviate this issue. The benefit is that using DMA to determine *M*_e_ lies in its simplicity and speed compared with alternative rheological methods, and it eliminates the need to fit data to the Williams–Landel–Ferry or Arrhenius equations.

**Figure 6:**
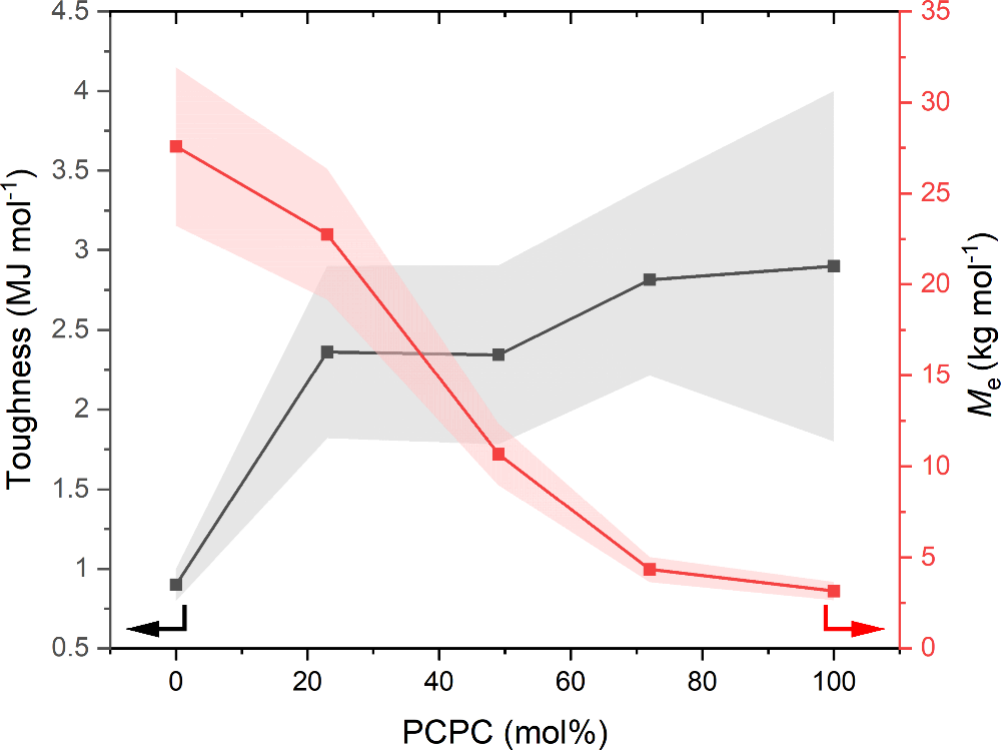
Plot showing tensile toughness and entanglement molecular weight of vs PCPC (mol %) for PCHC-*grad*-PCPC terpolymers.

Analysis of the entanglement molar mass for the different samples reveals that as the PCPC content increases, *M*_e_ decreases, consistent with increased chain entanglements. This finding is important since it helps to fine-tune the extent of molecular entanglement within these CO_2_-derived polycarbonates. In the same series, there was not any marked increase in tensile toughness as *M*_e_ decreases (Figure 6). Examining materials with steadily increasing PCPC contents from 23 to 72%, the tensile toughness measurements are within error of each other. This suggests that for all of these gradient terpolymers, the critical molecular mass (*M*_c_) was exceeded and chains are sufficiently entangled. While estimating the value of *M*_c_ as a multiple of *M*_e_ is somewhat controversial, most of these polymers show critical molar mass values, which appear to be in the range of 4.4*M*_e_ < *M*_c_ < 7.2*M*_e_—such ranges are observed for many other thermoplastics. Another aspect to emphasize is that despite the pure PCPC sample possessing a value of *M*_n_ which is 36 times greater than its *M*_e_, it remains easily processable and reprocessable at 150 °C.

Literature analysis reveals surprisingly few investigations where polymer structures were modified to control or minimize *M*_e_ and hence improve mechanical properties. However, unlike PCHC and PCPC, the previously investigated materials were usually applied at molar masses well above the *M*_c_ and the objective was usually to reduce *M*_e_ to improve processing.^45^ For example, Pawlak and co-workers controlled the *M*_e_ of poly(propylene) through the regulated slow cooling of a dilute polymer solution.^46^ The stress–strain profile for the PP, up to the yield point, was unaffected by the entanglement density, but the lower the *M*_e_, the faster the stress increased during the strain hardening stage. Bartczak controlled the *M*_e_ of ultrahigh molar mass poly(ethylene) through high-pressure annealing. Like Pawlak, he observed a greater strain hardening modulus and later onset of strain hardening with increased *M*_e_.^47^ Bartczak and co-workers also demonstrated that a lower entanglement density improves the properties of ultrahigh molar mass polypropylene.^48^ Hillmyer and co-workers showed that employing low *M*_e_ poly(γ-methyl-ε-caprolactone) as a central block in an ABA triblock copolymer is an effective means to produce tough thermoplastic elastomers.^49^ The elastomer tensile strength and extensibility were both controlled by the low entanglement molar mass polyester.

In this work, we have demonstrated that through incorporation of low entanglement molar mass carbonate segments into CO_2_ terpolymers, the elasticity of the thermoplastic polycarbonates are improved. It may be that this approach could be applied to toughen other sustainable plastics, many of which are well known to be highly brittle. Considering the epoxide comonomers, CPO and CHO are currently petrochemicals,^50^ but they can also be prepared by one-pot chemo-enzymatic reactions from triglycerides.^51^

**Figure 7:**
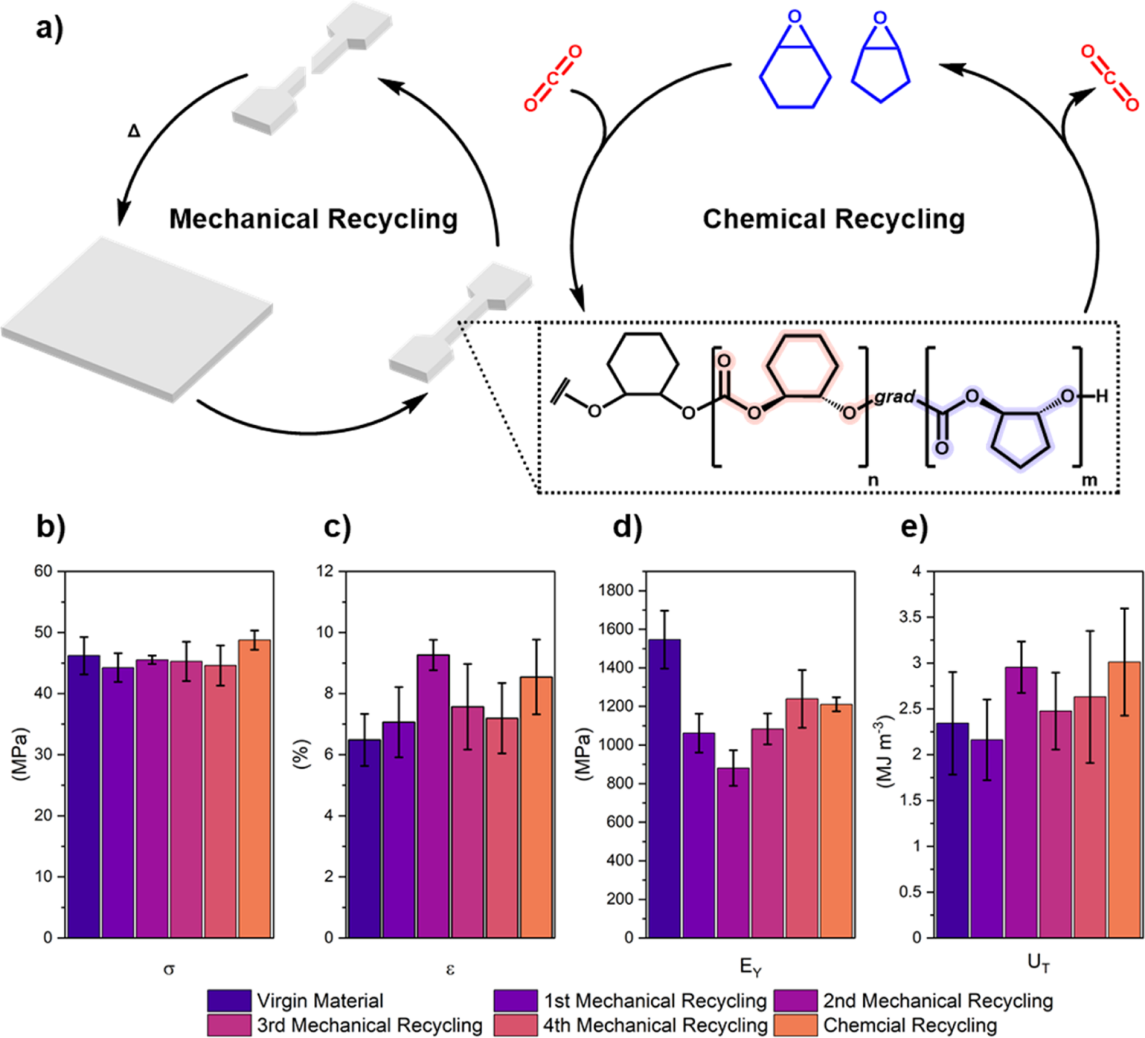
(a) Schematic of the mechanical property profiles of polycarbonate samples subjected to both mechanical and chemical recycling. (b) Tensile strength, (c) strain at break, (d) Young’s modulus, and (e) tensile toughness for virgin PCHC_0.51_-*grad*-PCPC_0.49_, after four mechanical reprocessing cycles and after chemical recycling to monomer and subsequent repolymerization. Mean values ± std. dev. From measurements conducted independently on five specimens.

The closed-loop recycling of thermoplastics is important to minimize end-of-life environmental impacts.^5,52^ To explore the mechanical recyclability of these gradient terpolymers, PCHC_0.51_-*grad*-PCPC_0.49_ was reprocessed four times by compression molding at 150 °C, 1.2 tons, 60 min. This allowed for the production of films suitable for uniaxial tensile testing (Figure 7 and Figure S15). Throughout mechanical recycling, the material remained colorless and transparent. SEC analysis, after each reprocessing cycle, revealed no change in polycarbonate molecular weight or dispersity, confirming its compatibility with the recycling conditions (Figure S16). The recycled products showed the same tensile strengths, elongations at break, and tensile toughness, over the four mechanical recycles, compared with virgin polymers. The slight decrease to Young’s modulus after the first reprocessing is proposed to be the result of minor contamination by dust/particulate matter into the film, which is more challenging to exclude on these smaller scales, resulting in defects that slightly reduce the stiffness of the material.

Finally, the ability to chemically recycle the gradient terpolymers to give the constituent epoxides CHO and CPO, together with CO_2_, was explored. A [LCoMg(OAc)_2_] recycling catalyst was previously reported for the highly efficient and fully selective solid-state PCHC depolymerization.^16^ It was, therefore, selected for investigation in the chemical recycling of the gradient terpolymers, and a recently reported TGA experimental method was used to monitor the chemical recycling reaction.^16^

Samples were prepared for these depolymerization experiments by solvent casting the polymer and catalyst mixtures into TGA crucibles (Scheme S1). In the experiments, the relative loadings of the [LCoMg(OAc)_2_] catalyst:polycarbonate repeat unit was 1:300. All depolymerizations were performed in triplicate at 140 °C, with an N_2_ flow rate of 25 mL min^–1^. The complete depolymerization of PCHC_0.51_-*grad*-PCPC_0.49_ and PCHC_0.28_-*grad*-PCPC_0.72_ was achieved. Plots of the polycarbonate mass loss against time were fit to exponentials, allowing determination of rate constants and, from these, turnover frequencies (TOFs) of 926 ± 14 and 1653 ± 201 h^–1^, respectively (Figure S17)_._ The depolymerization of PCHC_0.77_-*grad*-PCPC_0.23_ exhibited an initiation period (1 h) and showed a lower TOF of 270 ± 5 h^–1^. The lower rate is attributed to the higher absolute molar mass of that sample compared with others in the series. Previously, chain-end-capping experiments resulted in complete inhibition of the depolymerization and strongly supported a chain-end depolymerization mechanism.^16^ As such, longer chains have a lower chain-end-group concentration, which reduces their depolymerization rates relative to shorter chains. Moreover, the restricted chain motion of the longer polymer chains may reduce the mass transport to the catalyst, which would also slow the depolymerizations and results in the observed initiation period (Table S4).

The depolymerization rate constants (*k*_obs_) were determined through the exponential fits of the mass loss vs time data (*R*^2^ > 0.97, Figure S17). The depolymerization rate constants were 1.8 ± 0.2, 5.9 ± 1.0, and 9.4 ± 1.7 h^–1^ for PCHC_0.77_-*grad*-PCPC_0.23_, PCHC_0.51_-*grad*-PCPC_0.49_, and PCHC_0.28_-*grad*-PCPC_0.72_, respectively. These rate data match the TOF data, with PCHC_0.51_-*grad*-PCPC_0.49_ and PCHC_0.28_-*grad*-PCPC_0.72_ showing faster depolymerization than PCHC but slower than PCPC. PCHC_0.77_-*grad*-PCPC_0.23_ is slower than either of the homopolymers due to its higher molar mass and lower chain end concentration. To validate the TGA depolymerization experiments, a scaled-up depolymerization of PCHC_0.51_-*grad*-PCPC_0.49_ was conducted using 2.72 g of polymer and standard laboratory glassware. The terpolymer and catalyst were cast, as a film, into a glass vessel, and the resulting epoxides were collected under a partial vacuum (40 mbar, N_2_). The catalytic recycling resulted in the isolation of 1.41 g of a mixture of CHO:CPO with a 3:7 composition, as determined by ^1^H NMR spectroscopy. The overall recycling occurred with ∼77% isolated yield of the epoxides (Figure S18). One aspect to note is that the recycled epoxide ratio does not exactly match the starting terpolymer composition; instead, somewhat less CHO was isolated than expected. The difference can be understood by considering the chain-end depolymerization mechanism, which is initiated at the PCPC chain ends (see end-group titration experiments, Figure S7). As the depolymerization progresses, it is more challenging to ensure chain-catalyst interactions, which reduces the recovery of the CHO. Consequently, the overall PCPC and PCHC conversions were 95 and 47%, respectively.

To demonstrate that the monomer mixture could be effectively repolymerized, the CHO and CPO mixture was dried, over CaH_2_, distilled, and repolymerized (cat:monomer = 1:5000, 3 M toluene). The resulting gradient terpolymer, PCHC_0.29_-*grad*-PCPC_0.71_ (*M*_n_ = 98.1 kg mol^–1^, *Đ*_M_ = 1.31), had the expected composition and complete epoxide conversions were achieved (1.90 g, Figure S19 and S20). The isolated polycarbonate showed an overall chemical recycling yield of ∼70%. Uniaxial tensile testing of the chemically recycled polycarbonate showed the same mechanical properties as the starting material and to the mechanically recycled polymers (Figure 7, Table S5, and Figure S21). These findings suggest that in the future both mechanical and chemical recycling would be feasible for these terpolymers.

## Conclusions

A series of high molar mass, gradient polycarbonates, PCHC-*grad*-PCPC, were produced using CO_2_/epoxide ROCOPs. The materials overcame the brittleness and processing issues associated with prior investigations of poly(cyclohexene carbonate) and exemplified the benefits of cyclopentene carbonate linkage incorporation. The terpolymerization of cyclohexene oxide, cyclopentene oxide, and CO_2_, using a high activity and selectivity Zn(II)Mg(II) catalyst, formed amorphous gradient polycarbonates in which both carbonate segments were miscible. All terpolymers showed high molar masses (*M*_n_ > 85 kg mol^–1^), glass transition temperatures (*T*_g_ > 96 °C), and wide processing temperature ranges (>145 °C). The gradient terpolymers showed lower entanglement molar masses than PCHC (4 < *M*_e_ < 23 kg mol^–1^), and all samples showed molar mass values that should exceed the critical molar mass. The gradient terpolymers exhibit significantly greater elongation at break and tensile strength than PCHC, resulting in a 200% increase in the tensile toughness. The polycarbonates were mechanically recycled via compression molding at 150 °C up to four times without any discoloration or loss in mechanical performance. They were also efficiently chemically recycled to epoxide/CO_2_, using a solid-state process and Co(II)Mg(II) catalyst. The isolated monomers were used to make a recycled polycarbonate which showed properties equivalent to those of the starting materials. The incorporation of some PCPC linkages improves the properties of PCHC. Overall, this work highlights the potential to apply terpolymerizations to improve the properties of CO_2_-derived thermoplastics.

## Data Availability

The data that support the findings of this study are available from the corresponding author upon reasonable request.

## Supporting Information Available

Experimental and instrument details, polymer synthesis, characterization, and additional mechanical property measurements (PDF)
