# Supplementary material for: Controlled Carbon Dioxide Terpolymerizations to Deliver Toughened yet Recyclable Thermoplastics
Source: Macromolecules. 2024 Apr 24;57(9):4199–207. doi: 10.1021/acs.macromol.4c00455 (PMC11100004; doi:10.1021/acs.macromol.4c00455)
Supplement: Supplementary file 1 — ma4c00455_si_001.pdf [file ma4c00455_si_001.pdf]

Supporting Information:

Controlled Carbon Dioxide Terpolymerizations to Deliver Toughened Yet Recyclable Thermoplastics

*Kam C. Poon, Madeleine L. Smith and Charlotte K. Williams**

Chemistry Research Laboratory, Department of Chemistry, University of Oxford, Oxford, OX1 3TA, U.K.

E-mail: charlotte.williams@chem.ox.ac.uk

Contents

Experimental Details 4

Scheme S1: Solid-state PCHC-*grad*-PCPC depolymerization set up for TGA.[2] 5

Reagents and Methods 6

Table S1: Summary of Polymerization Conditions and Results 6

Figure S1: Representative ^{1}H NMR spectrum (400 MHz, $CDCl_3$) of PCHC-*grad*-PCPC terpolymer ($PCHC_{0.51}$-*grad*-$PCPC_{0.49}$). 8

Figure S2: Representative ^{1}H COSY NMR spectrum (400 MHz, $CDCl_3$) of PCHC-*grad*-PCPC terpolymer ($PCHC_{0.51}$-*grad*-$PCPC_{0.49}$). 8

Figure S3: Representative ^{1}H – ^{13}C HSQC NMR spectrum (400 MHz, $CDCl_3$) of PCHC-*grad*-PCPC terpolymer ($PCHC_{0.51}$-*grad*-$PCPC_{0.49}$). 9

Figure S4: Representative $^{13}C\{^{1}H\}$ HSQC NMR spectrum (400 MHz, $CDCl_3$) of PCHC-*grad*-PCPC terpolymer ($PCHC_{0.51}$-*grad*-$PCPC_{0.49}$). 9

Figure S5: SEC (THF, 1 mL min^{-1}) traces for the PCHC-*grad*-PCPC terpolymers. The instrument is calibrated with poly(styrene) standards. 10

Figure S6: Representative ^{1}H DOSY NMR spectrum (500 MHz, $CDCl_3$) of PCHC-*grad*-PCPC terpolymer ($PCHC_{0.51}$-*grad*-$PCPC_{0.49}$). 10

Table S2: Fineman-Ross Analysis Results of CPO/CHO with CO_2 Terpolymerization 11

Figure S7: $^{31}P\{^{1}H\}$ NMR spectra used for end-group analysis of PCHC, PCHC-*grad*-PCPC terpolymers and PCPC. 11

Figure S8: Differential Scanning Calorimetry (DSC) data for PCHC-*grad*-PCPC terpolymers. 12

Figure S9: Thermogravimetric Analysis (TGA) data for PCHC-*grad*-PCPC terpolymers. 12

Table S3: DMTA Results Used to Determine Entanglement Molecular Weight 13

Figure S10: Dynamic mechanical temperature analysis (DMTA) temperature sweeps for PCHC (Table S3). 14

Figure S11: Dynamic mechanical temperature analysis (DMTA) temperature sweeps for $PCHC_{0.77}$-*grad*-$PCPC_{0.23}$ (Table S3). 15

Figure S12: Dynamic mechanical temperature analysis (DMTA) temperature sweeps for $PCHC_{0.51}$-*grad*-$PCPC_{0.49}$ (Table S3). 16

Figure S13: Dynamic mechanical temperature analysis (DMTA) temperature sweeps for $PCHC_{0.28}$-*grad*-$PCPC_{0.72}$ (Table S3). 17

Figure S14: Dynamic mechanical temperature analysis (DMTA) temperature sweeps for PCPC (Table S3). 18

Figure S15: Photographs of $PCHC_{0.51}$-*grad*-$PCPC_{0.49}$ throughout the compression moulding and mechanical recycling process. Film thickness ~0.2 mm. 19

Figure S16: SEC (THF, 1 mL min^{-1}) traces for the $PCHC_{0.51}$-*grad*-$PCPC_{0.49}$ after each cycle of mechanical reprocessing. The SEC instrument is calibrated with poly(styrene) standards. 19

Figure S17: Solid-state depolymerization data for $PCHC_{0.51}$-*grad*-$PCPC_{0.49}$ terpolymers using [$LCoMg(OAc)_2$] catalyst (1:300), at 140 ˚C. Plots show terpolymer mass loss data vs. time. The data is fit to exponentials to determine the pseudo first order rate constants, k_{obs}. 20

Table S4: Data for the terpolymer depolymerizations using the Co(II)Mg(II) Catalyst 20

Figure S18: ^{1}H NMR spectrum (400 MHz, $CDCl_3$) of the CHO and CPO isolated from the depolymerization. The relative ratio, indicated by the integrals, is CHO:CPO = 73 : 27. 21

Figure S19: ^{1}H NMR spectrum (400 MHz, $CDCl_3$) of the chemically recycled (i.e. re-polymerized) PCHC-*grad*-PCPC terpolymer. 21

Figure S20: SEC (THF, 1 mL min^{-1}) traces for the $PCHC_{0.51}$-*grad*-$PCPC_{0.49}$ and chemically recycled $PCHC_{0.29}$-*grad*-$PCPC_{0.71}$. 22

Table S5: Data for the Chemically Recycled PCHC-*grad*-PCPC Terpolymers 23

Figure S21: Representative stress-strain data (10 mL min^{-1}) for $PCHC_{0.51}$-*grad*-$PCPC_{0.49}$ after repeated cycles of mechanical reprocessing and chemical recycling. 24

References 25

Experimental Details

NMR Spectroscopy. ^{1}H and $^{31}P\{^{1}H\}$ NMR spectra were obtained using a Bruker AVIII HD 400 NMR spectrometer. $^{13}C\ \{^{1}H\}$ NMR spectra were obtained using a Bruker Avance III AVD500 NMR spectrometer. ^{1}H DOSY spectra were obtained using a Bruker NEO600 NMR spectrometer.

Size Exclusion Chromatography (SEC). Polymers (2-5 mg) dissolved in THF. Samples were passed through 0.2 µm PTFE filters prior to analysis. Analysis was carried out on a Shimadzu LC-20AD instrument, equipped with a Refractive Index (RI) detector and two PSS SDV 5 µm linear M columns. HPLC grade THF was used as the eluent at 1.0 mL/min at 30 °C. Monodisperse polystyrene standards were used for calibration.

Phosphorus End-Group Tests. Polymer samples (40 mg) were dissolved in $CDCl_3$ (0.5 mL) and a solution (40 µL) containing $Cr(acac)_3$ (5.5 mg) and internal standard, bisphenol A (400 mg) in pyridine (10 mL), followed by 40 µL of 2-chloro-4,4,5,5-tetramethyl dioxaphospholane.[1]

Differential Scanning Calorimetry (DSC). Recorded for purified polymer samples of homopolymers and terpolymers were measured using a DSC25 (TA Instruments). A sealed, empty crucible was used as a reference, and the DSC was calibrated using sapphire and indium. Samples were heated from -80 °C to 150 °C, at a rate of 10 °C min^{-1} under N_2 flow (80 mL min^{-1}) followed by a 5-minute isotherm at 150 °C to erase thermal history. Samples were subsequently cooled to -80 °C, at a rate of 10 °C min^{-1}, and kept at -80 °C for a further 5 minutes, followed by a heating-cooling procedure from -80 °C to 150 °C, at a rate of 10 °C min^{-1}. Each sample was analysed over two heating-cooling cycles. Glass transition temperatures (T_g) are reported as the midpoint of the transition taken from the second heating cycle.

Thermogravimetric Analysis (TGA). Measured using a TGA5500 system (TA Instruments). Samples were heated from 30 °C to 600 °C, at a rate of 5 °C min^{-1}, under N_2 flow (100 cm^3 min^{-1}).

Film Preparation. Transparent films were prepared by solvent casting into Teflon moulds from THF. Films were dried in a vacuum oven for at least 48 hours prior to use. Samples were then compression moulded using a Carver mini CH CE Press (5420CE.4040C00) with heated plates and hydraulic compression press. Polymers were placed between two metal sheets lined with Teflon, heated to 150 °C, for 15 min, under no pressure, then a further 60 min, under 1.2 ton m^{-2} and allowed to cool overnight.

Tensile Testing. Tests were carried out using an Instron 8600 series universal testing system, using a 50 N load cell and 250 N pneumatic grips. Dumbbell-shaped specimens were cut using a Zwick ZCP020 cutting press, equipped with a cutting device for ISO 527-2 type 5B. Uniaxial extension experiments (10 mm min^{-1} cross-head speed) were run according to ISO 527.

Dynamic Mechanical Analysis (DMA). Thermal analysis (DMTA) was carried out using a DMA850 (TA Instruments), using an ACS III cooling system. Specimens of uniform width (5.3 mm) were cut using two parallel blades. Samples were heated from -80 °C to 250 °C (or until the material deformed beyond the limits of the geometry employed), at a rate of 3 °C min^{-1}, with a frequency of 1 Hz, 0.1 N pre-load force and 0.1 % strain amplitude.

Solid-State PCHC Depolymerization. The chemical recycling experiments were conducted using the previously reported procedure, conducted in a TGA instrument.[2] The sample was prepared as followed: in the glovebox, PCHC-*grad*-PCPC (1.00 mmol) was dissolved in THF (1 mL). The Mg(II)Co(II) catalyst (0.3 mmol) was added to the vial. The catalyst:polymer stock-solution (40 µL) was transferred to an aluminium TGA crucible. The crucible was placed under vacuum, for 30 minutes, before being crimped in the glovebox with a hermetic seal. The crucible was then transferred to a TGA instrument for solid-state depolymerization using the method outlined below.

1. N_2 flow of 25.0 mL min^{-1}
2. Equilibrate at 30 °C
3. Heat to 140 °C
4. Isotherm at 140 °C, whilst monitoring mass loss
5. After 3 h (> 95 % mass loss in all cases), sample was cooled to 30 °C

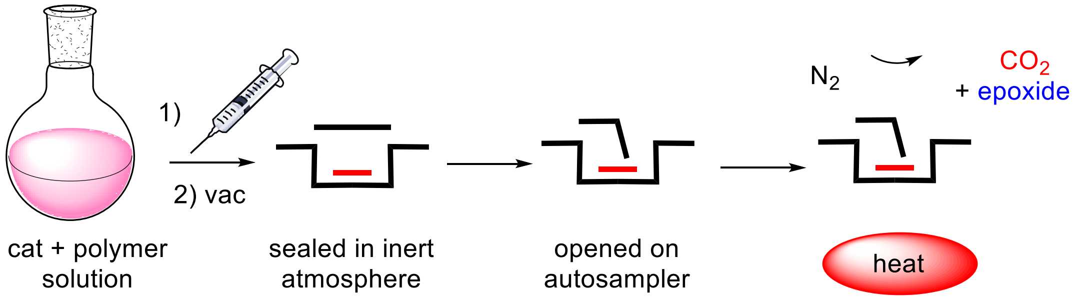

Scheme S1: Solid-state PCHC-*grad*-PCPC depolymerization set up for TGA.[2]

Laboratory-Scale Chemical Recycling. Under an N_2 atmosphere, PCHC-*grad*-PCPC (2.72 g, 20 mmol), dissolved in THF (3 mL), was added to a Schlenk tube containing the Mg(II)Co(II) catalyst (51 mg, 0.07 mmol). The Schlenk tube was placed under dynamic vacuum, overnight, to remove the THF and form a film on the glass walls of the vessel. A distillation glassware apparatus (short-path) was attached to it, with the collection flask being cooled. The apparatus was placed under vacuum (~40 mbar) and the Schlenk tube heated to 140 °C for 8 h, after which atmospheric pressure was re-established in the system using N_2.

The collected epoxide (1.51 g, 85 % yield) was distilled over CaH_2 and then repolymerised with a [$LZnMg(C_6F_5)_2$] catalyst, (catalyst:epoxides = 1:5000, 3 M epoxide solution in toluene, 80 °C, 40 bar CO_2, 5 days).

Reagents and Methods

The macrocyclic ligand, H_2L, was synthesized following a previously reported procedure.[2] Magnesium bis(1,1,1,3,3,3-hexamethyldisilazan-2-ide) (97%) and bis(pentafluorophenyl)zinc (97%) were purchased from Sigma-Aldrich. Bis(pentafluorophenyl)zinc was used as received and magnesium bis(1,1,1,3,3,3-hexamethyldisilazan-2-ide) was recrystallized from hexane. Solvents used for synthesis and polymerization were collected from a solvent purification system (SPS), degassed with three freeze-pump-thaw cycles, and stored over 4 Å molecular sieves, under an inert atmosphere. Cyclohexene 1,2-epoxide (98 %) (CHO) was purchased from Alfa Aesar, dried by stirring over CaH_2, followed by fractional distillation at 60 °C. Cyclopentene 1,2-epoxide (98 %) (CPO) was purchased from Sigma-Aldrich, dried by stirring over CaH_2, followed by fractional distillation at 60 °C.

Synthesis of [LZnMg(C_6F_5)$_2$] Catalyst[3]

The catalyst was synthesized following a previously reported procedure.[3] Under inert conditions, the macrocyclic ligand (H_2L) (0.50 g, 0.90 mmol) and recrystallised Mg{N[Si(CH_3)$_3$]$_2$}$_2$ (0.31 g, 0.90 mmol) were dissolved in THF (10 mL), at 25 °C, for 1 hour. Zn(C_6F_5)$_2$ was dissolved in THF (5 mL) and added dropwise to the reaction mixture. The dark orange solution was stirred overnight, at 25 °C. The pale orange reaction mixture was then cooled to -29 °C and the supernatant solvent was removed. The solid product was washed (hexane) and the product (precipitate) dried under reduced pressure. Yield: 0.54 g (61 %). ^{1}H NMR (400 MHz, $CDCl_3$) δ 6.81 (s, 2H), 6.76 (s, 2H), 4.42 – 4.25 (m, 4H), 3.40 (d, J = 13.7 Hz, 2H), 3.26 (d, J = 13.3 Hz, 2H), 3.14 – 2.94 (m, 4H), 2.68 (s, 6H), 2.11 – 2.00 (m, 2H), 1.27 (s, 3H), 1.20 (s, 18H), 1.17 (s, 3H), 1.06 – 1.01 (m, 6H).

Synthesis of [LCoMg(OAc)$_2$] Catalyst[4]

Mg(N(SiMe_3)$_2$)$_2$ (0.44 g, 0.13 mmol) and Co(OAc)$_2$ (0.23 g, 0.13 mmol) was added to a solution of H_2L (0.70 g, 0.13 mmol) in THF (6 mL) and heated to 100 °C in a sealed Schlenk tube for 16 h. The solvent was removed, in vacuo, and the solid washed with pentane (3 x 10 mL) to afford the product as a pale pink solid. Yield: 0.41 g (43 %). MS (MALDI-ToF): m/z 692.31 [LMgCo(OAC)]$^+$.

General Procedure for CHO, CPO, CO_2 Terpolymerization

[LZnMg(C_6F_5)$_2$] (5 mg, 0.01 mmol), cyclohexane-1,2-diol (2.4 mg, 0.02 mmol), cyclohexene oxide (2.60 mL, 25 mmol) and cyclopentene oxide (2.18 mL, 25 mmol) were dissolved in toluene (10 mL). The solution was added into a high-pressure Steel reactor which was placed under 40 bar of CO_2 (using a triple manifold line), heated to 80 °C and stirred for 96 h. The reaction mixture was quenched by exposed to air and the polymer was precipitated in methanol (3x) and dried under reduced pressure.

Table S1: Summary of Polymerization Conditions and Results

cat. /mg	CHD /mg	CHO /mL	CPO /mL	Toluene /mL	CO_2 /bar	CHO conv./ %	CPO conv./ %	PCHC :PCPC
5	2.4	2.60	2.18	10	20	86	58	71:29
5	2.4	2.60	2.18	10	40	98	93	51:49
5	2.4	3.89	1.12	10	40	99	91	77:23
5	2.4	1.30	3.36	10	40	96	90	28:72

Equations

$$G^0{}_N = \frac{4\rho RT}{5M_e} \quad \text{(S1)}$$

Where G^0_N is the rubbery plateau modulus (at tan(δ) minimum), ρ is the polymer density (ρ ranges from 800–1100 kg·m^{-3} in most estimates),[5] R is the ideal gas constant, T is the temperature and M_e is the entanglement molecular weight.

$$\frac{(f-1)}{F} = r_{CPO} - r_{CHO}\frac{f}{F^2} \quad \text{(S2)}$$

Where f is the ratio of monomers enchained in the terpolymer, F is the feed ratio of monomers, r_{CPO} is the reactivity ratio of CPO and r_{CHO} is the reactivity ratio of CHO.

$$\frac{1}{T_{g,theo}} = \frac{w_{PCHC}}{T_{g,PCHC}} + \frac{w_{PCPC}}{T_{g,PCPC}} \quad \text{(S3)}$$

Where $T_{g,theo}$ is the theoretical T_g of the terpolymer, w_{PCHC} and w_{PCPC} are the weight fractions of PCHC and PCPC respectively, and $T_{g,PCHC}$ and $T_{g,PCPC}$ are the glass transition temperatures for PCHC and PCPC.

$$E_N^0 = 2G_N^0(1+\nu) \quad \text{(S4)}$$

Where E^0_N is the elastic modulus, G^0_N is the sheer modulus and ν is the Poisson ratio (assumed to be 0.36 for all materials).[6]

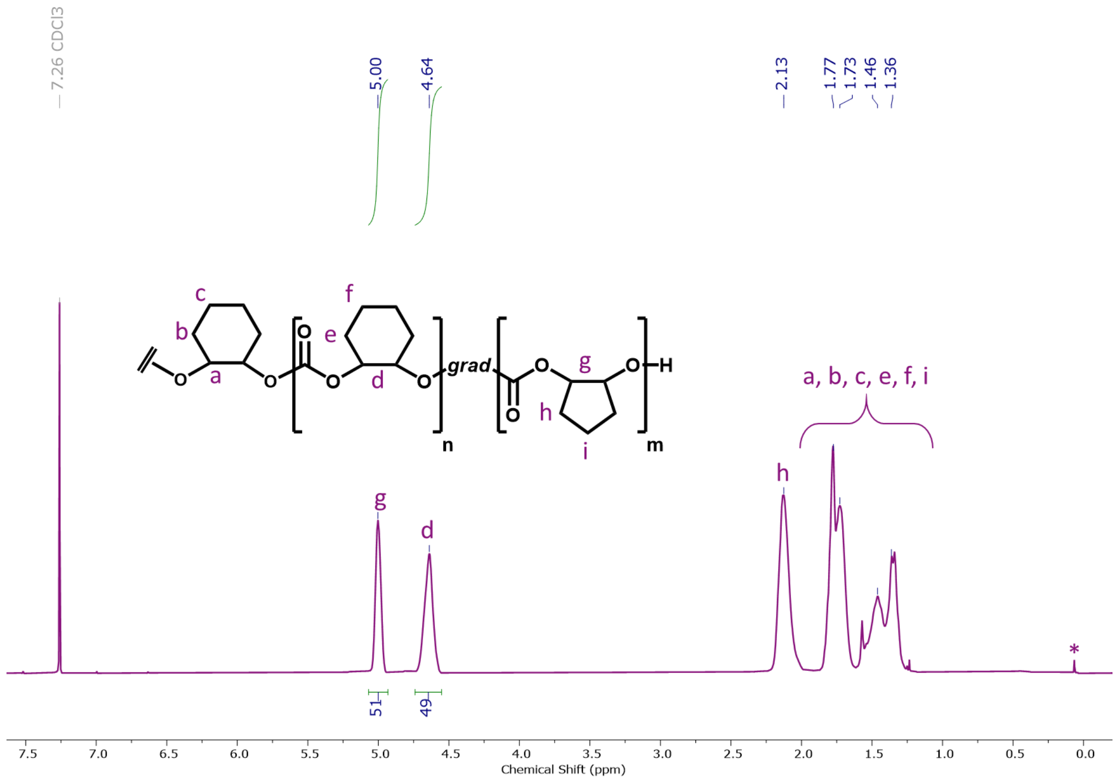

Figure S1: Representative ^{1}H NMR spectrum (400 MHz, $CDCl_3$) of PCHC-*grad*-PCPC terpolymer ($PCHC_{0.51}$-*grad*-$PCPC_{0.49}$).

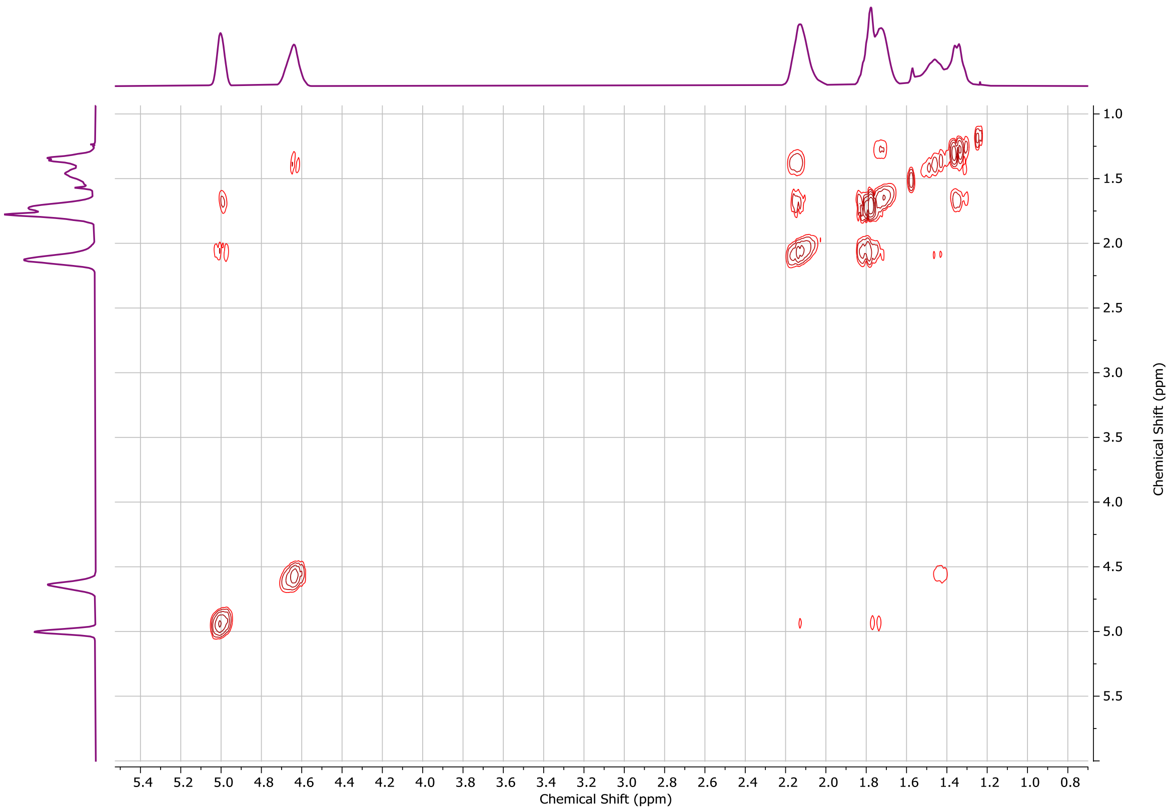

Figure S2: Representative ^{1}H COSY NMR spectrum (400 MHz, $CDCl_3$) of PCHC-*grad*-PCPC terpolymer ($PCHC_{0.51}$-*grad*-$PCPC_{0.49}$).

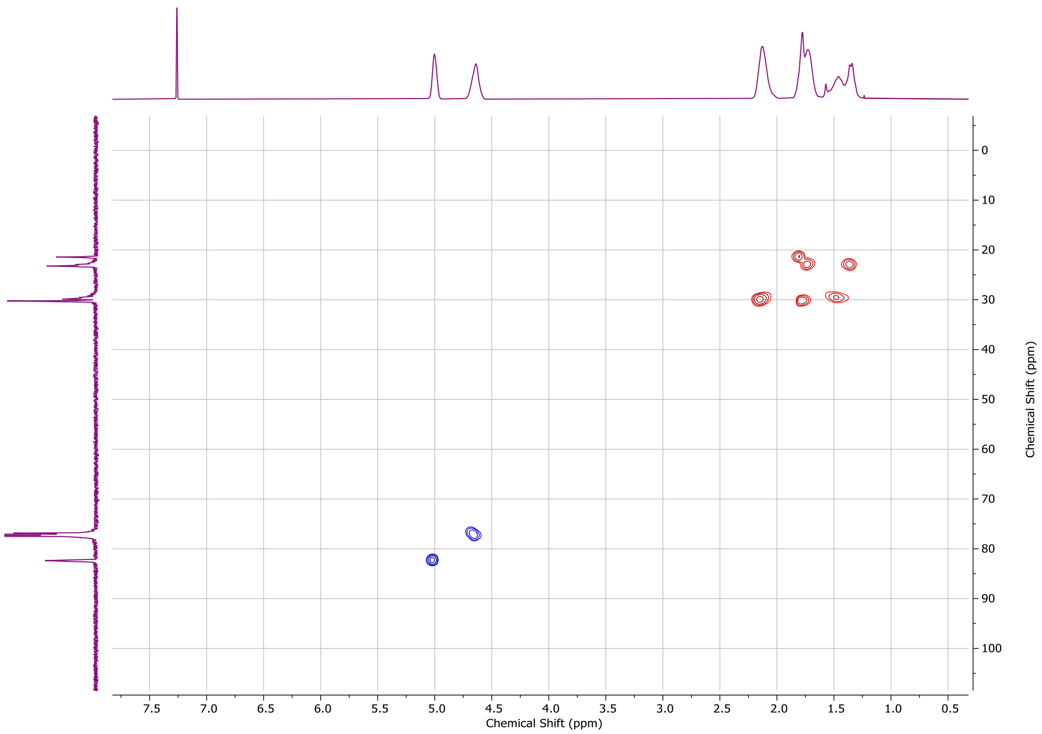

Figure S3: Representative ^{1}H – ^{13}C HSQC NMR spectrum (400 MHz, $CDCl_3$) of PCHC-*grad*-PCPC terpolymer ($PCHC_{0.51}$-*grad*-$PCPC_{0.49}$).

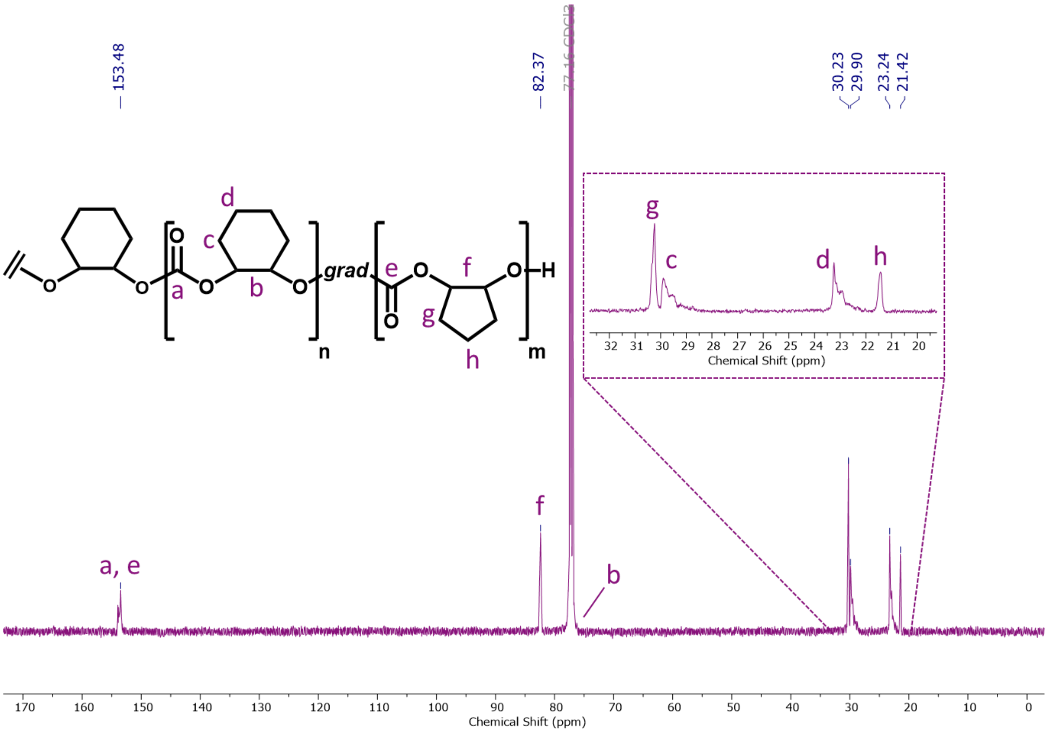

Figure S4: Representative$^{13}C\{^{1}H\}$ HSQC NMR spectrum (400 MHz, $CDCl_3$) of PCHC-*grad*-PCPC terpolymer ($PCHC_{0.51}$-*grad*-$PCPC_{0.49}$).

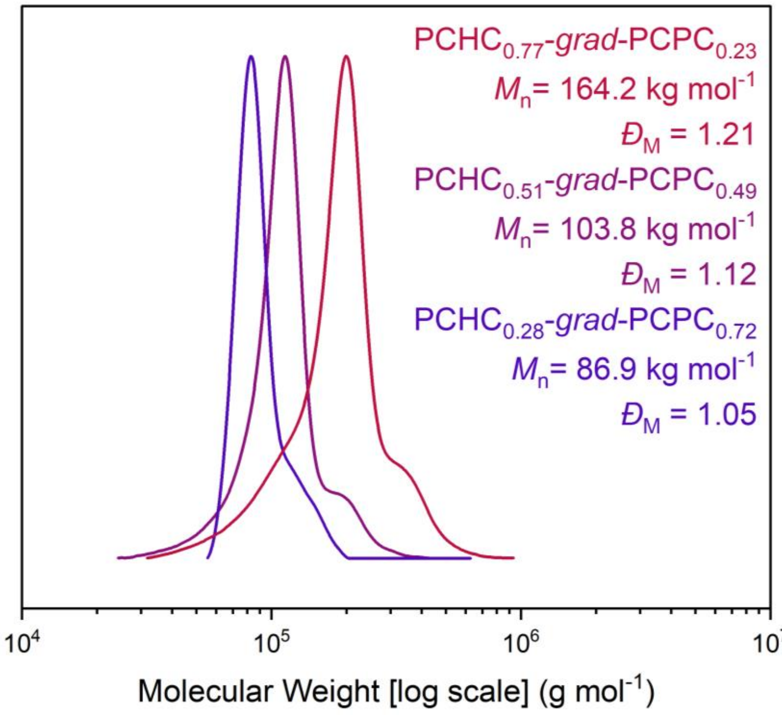

Figure S5: SEC (THF, 1 mL min^{-1}) traces for the PCHC-*grad*-PCPC terpolymers. The instrument is calibrated with poly(styrene) standards.

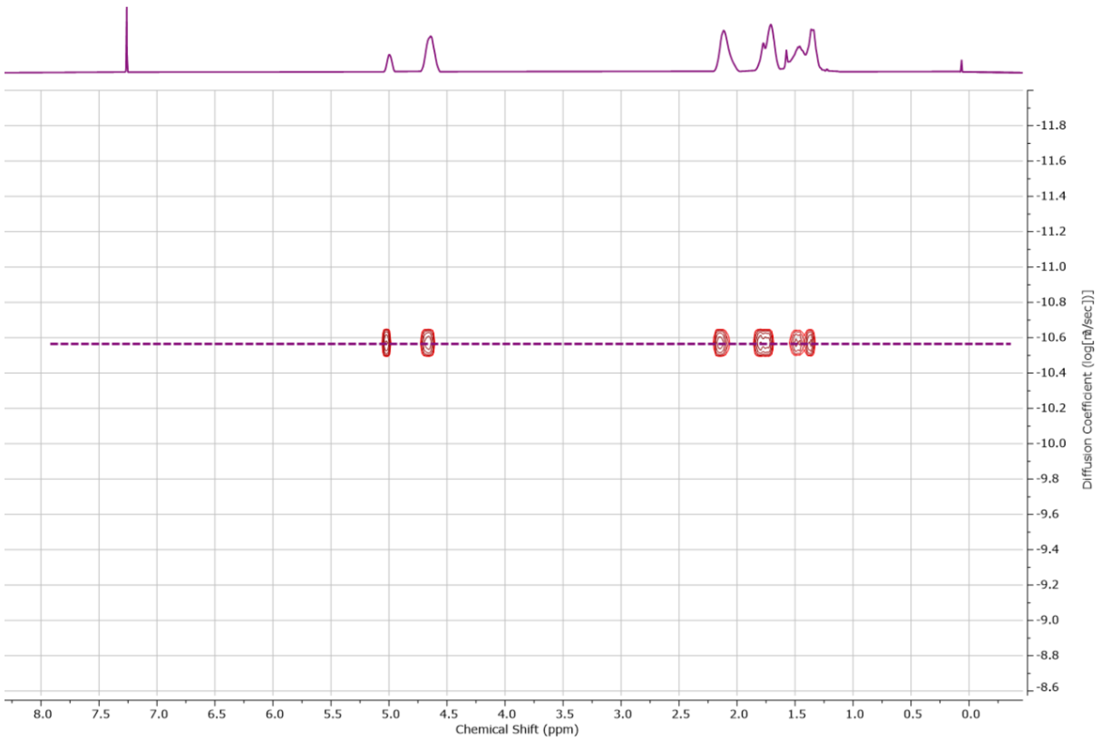

Figure S6: Representative ^{1}H DOSY NMR spectrum (500 MHz, $CDCl_3$) of PCHC-*grad*-PCPC terpolymer ($PCHC_{0.51}$-*grad*-$PCPC_{0.49}$).

Table S2: Fineman-Ross Analysis Results of CPO/CHO with CO_2 Terpolymerization

Entry	F(CPO/CHO) in feed	f(CPO/CHO) in polymer	f/F^2	(f-1)/F
1	0.56	0.24	0.77	-1.36
2	1.43	0.40	0.20	-0.42
3	1.14	0.46	0.35	-0.47
4	3.93	2.21	0.14	0.31
5	0.33	0.19	1.74	-2.45
6	0.54	0.24	0.82	-1.41
7	0.43	0.24	1.30	-1.77

Reactions were performed at 80 °C, 1 bar CO_2, [cat.]:[CHD]:[CPO+CHO] = 1:4:1000, [CPO+CHO] = 5 M toluene and stopped after 2 h.

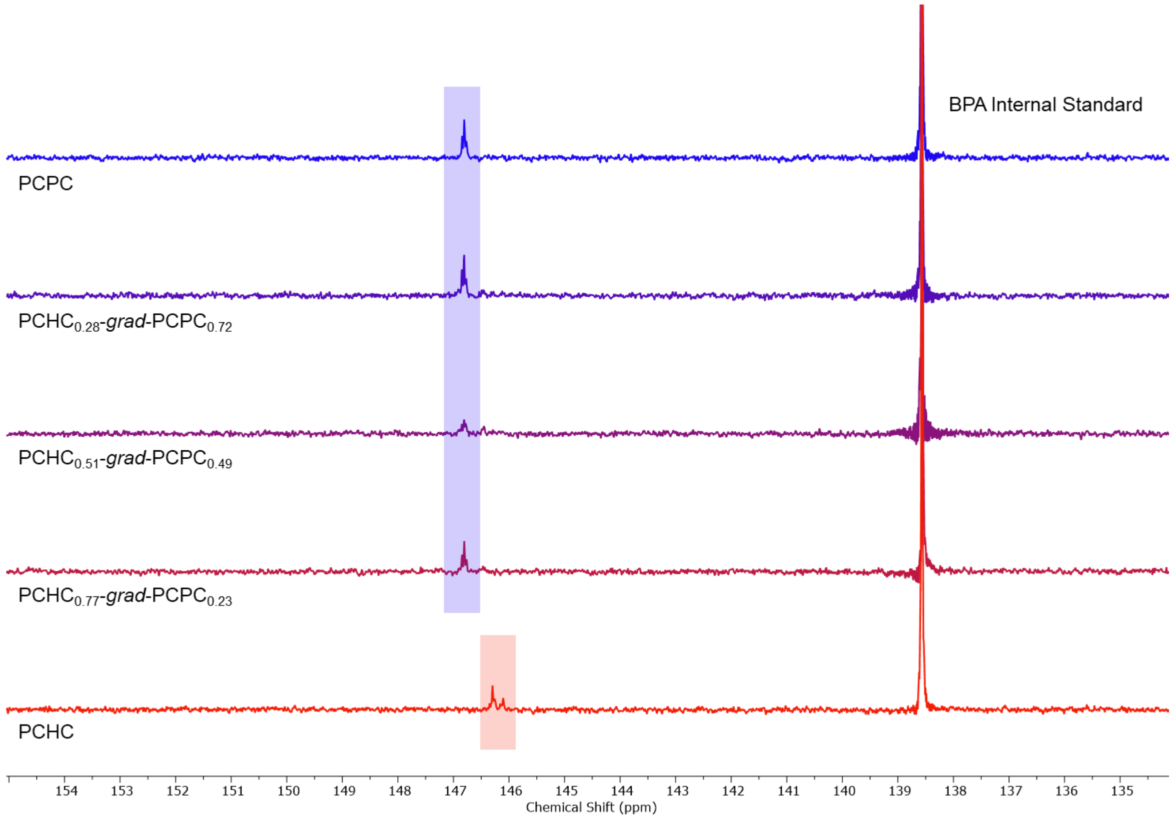

Figure S7: $^{31}P\{^1H\}$ NMR spectra used for end-group analysis of PCHC, PCHC-*grad*-PCPC terpolymers and PCPC.

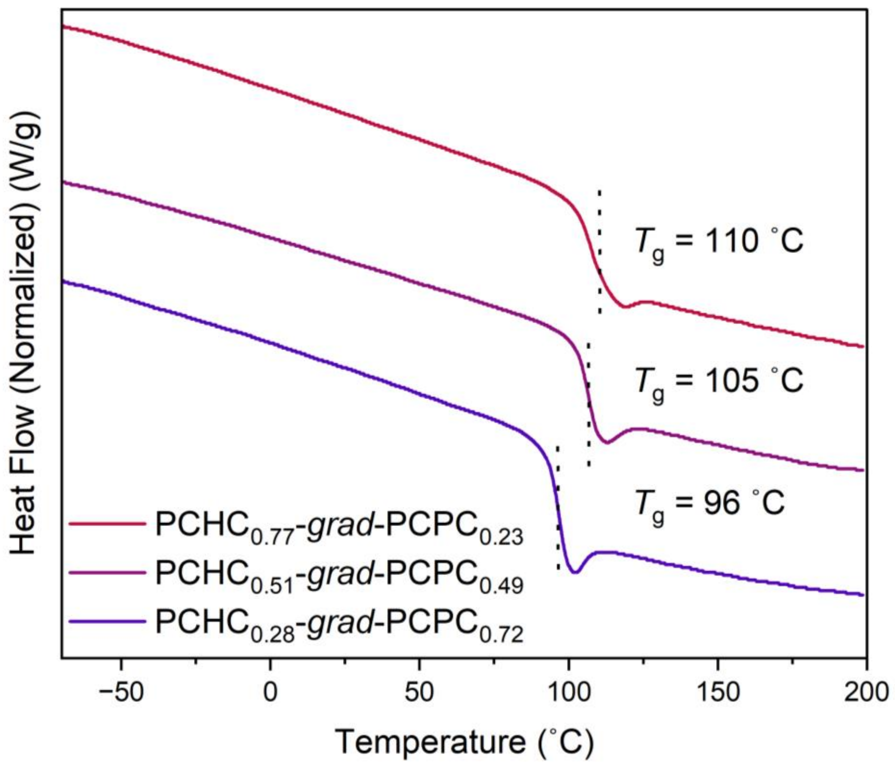

Figure S8: Differential Scanning Calorimetry (DSC) data for PCHC-*grad*-PCPC terpolymers.

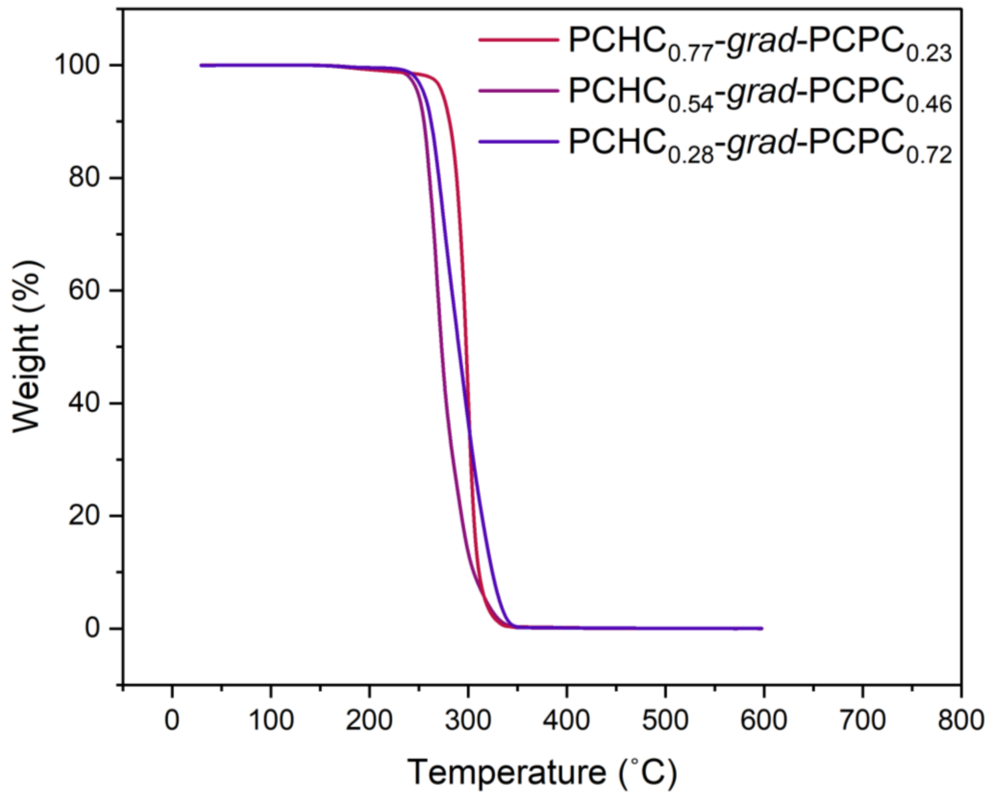

Figure S9: Thermogravimetric Analysis (TGA) data for PCHC-*grad*-PCPC terpolymers.

Table S3: DMTA Results Used to Determine Entanglement Molecular Weight

Polymer	E^{0}_{N} /MPa	T /K
PCHC	0.26933	432.15
$PCHC_{0.77}$-*grad*-$PCPC_{0.23}$	0.32828	434.54
$PCHC_{0.77}$-*grad*-$PCPC_{0.23}$	0.64471	400.46
$PCHC_{0.77}$-*grad*-$PCPC_{0.23}$	1.55958	393.98
PCPC	2.06587	379.62

Values of E^{0}_{N} and T taken from the minimum tan (δ) in the plateau region. The value for the density, ρ = 1135 kg·m^{-3} for PCHC.[7] For the terpolymers and PCPC, a density range of 800–1100 kg·m^{-3} was used to provide an estimate.[7]

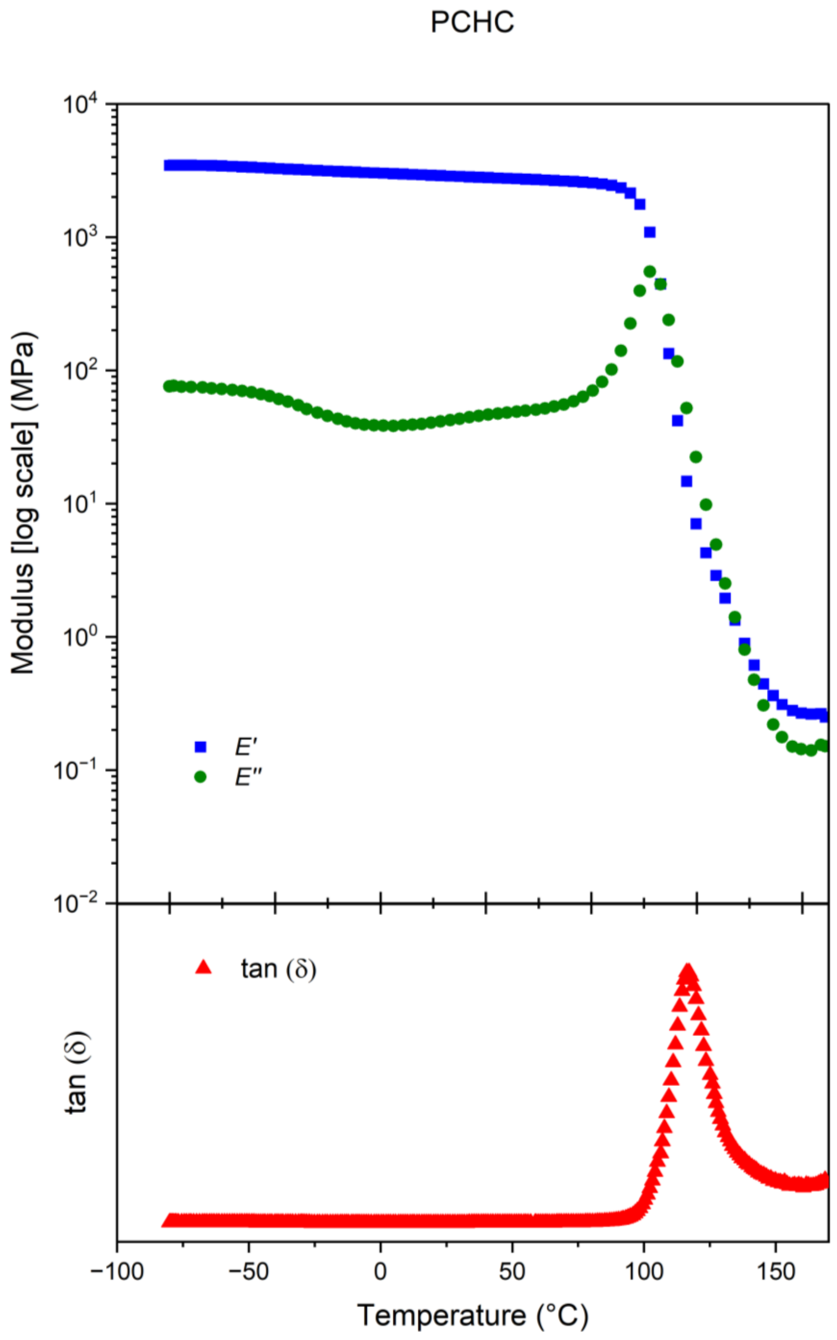

Figure S10: Dynamic mechanical temperature analysis (DMTA) temperature sweeps for PCHC (Table S3).

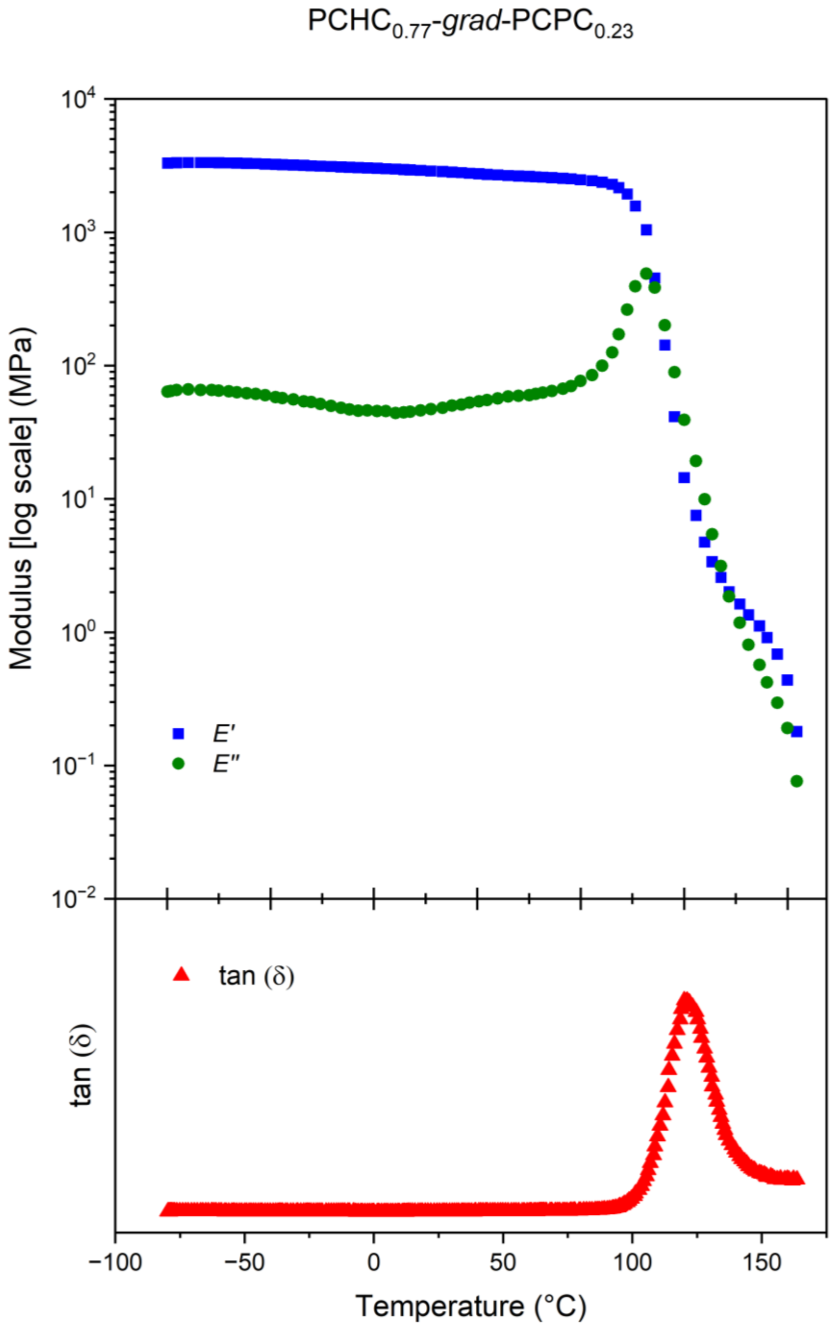

Figure S11: Dynamic mechanical temperature analysis (DMTA) temperature sweeps for $PCHC_{0.77}$-*grad*-$PCPC_{0.23}$ (Table S3).

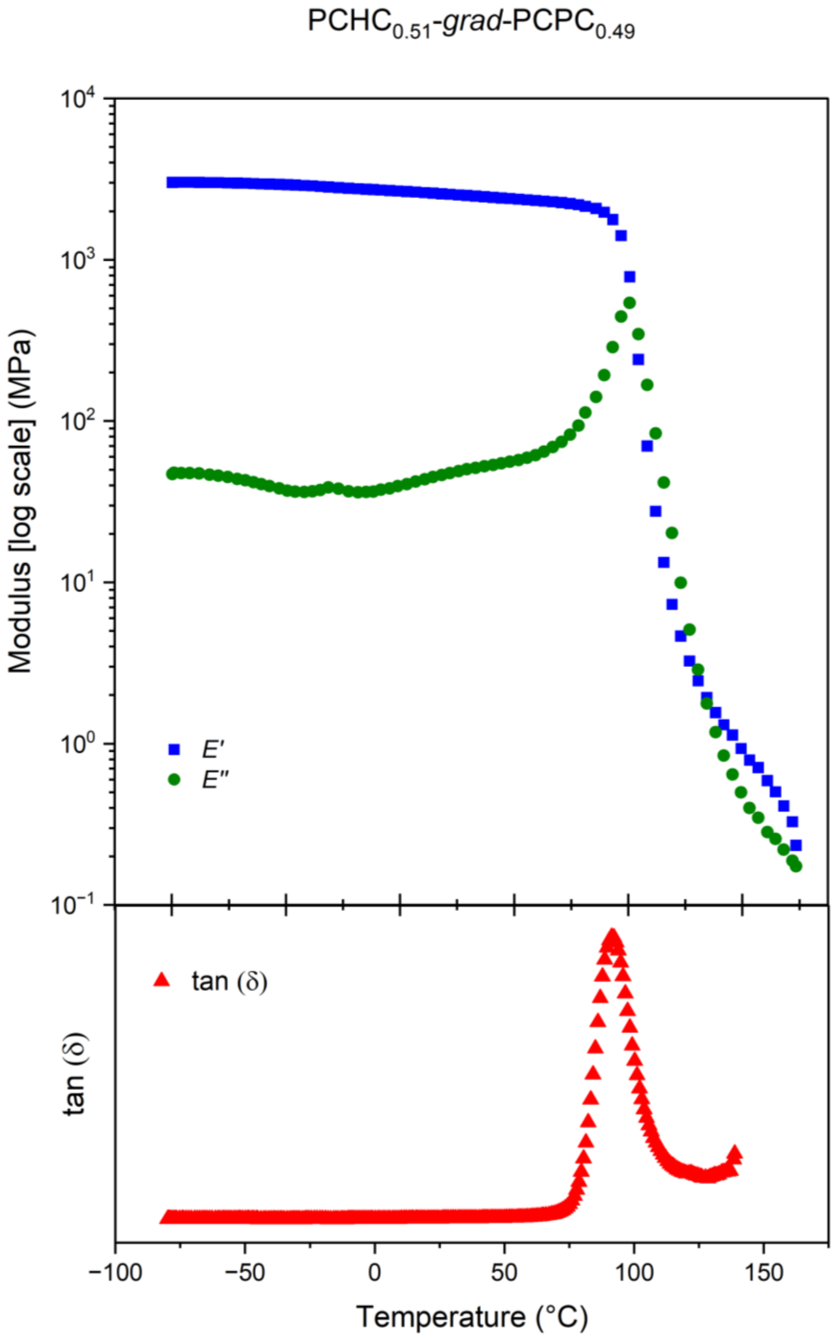

Figure S12: Dynamic mechanical temperature analysis (DMTA) temperature sweeps for $PCHC_{0.51}$-*grad*-$PCPC_{0.49}$ (Table S3).

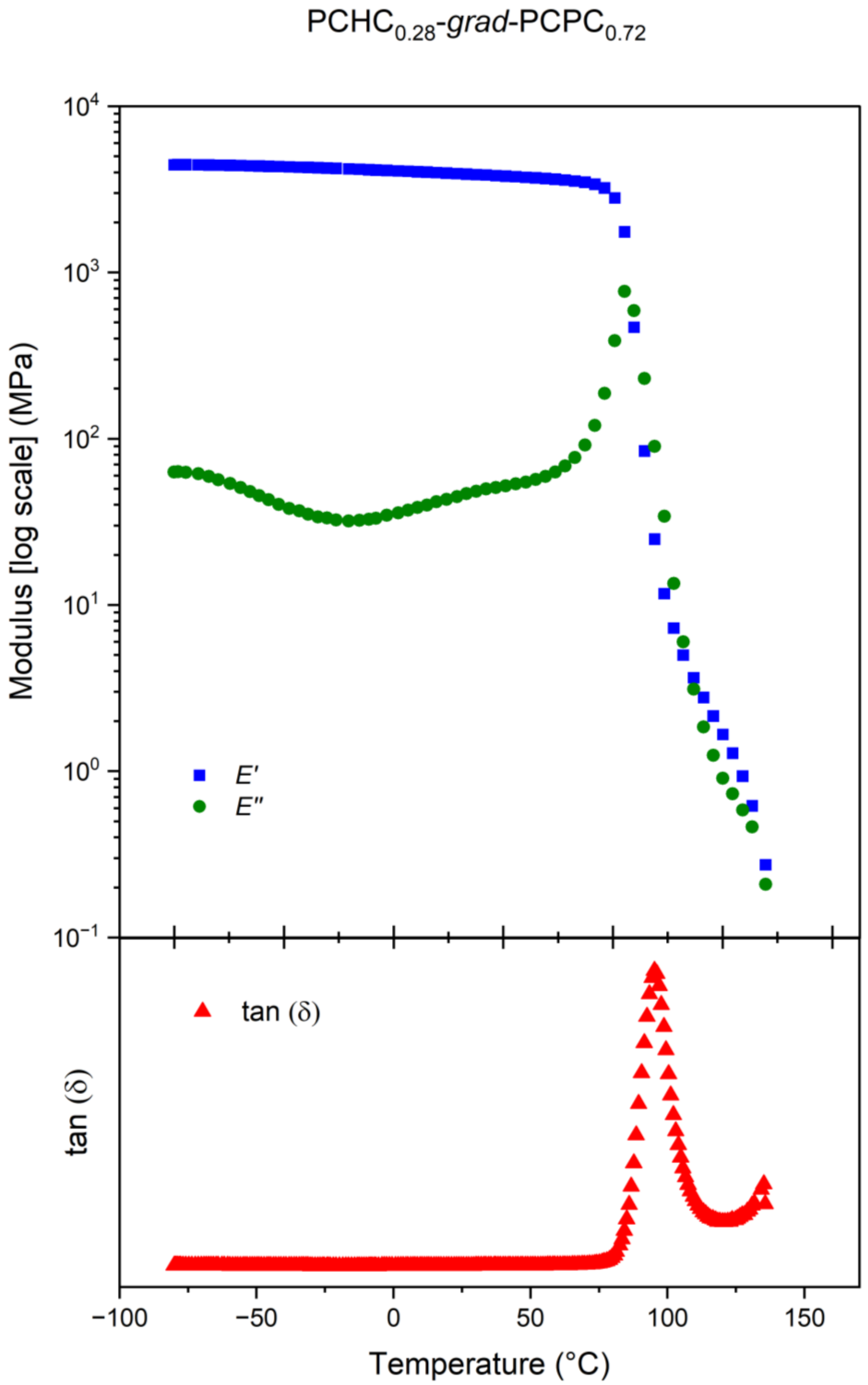

Figure S13: Dynamic mechanical temperature analysis (DMTA) temperature sweeps for PCHC$_{0.28}$-*grad*-PCPC$_{0.72}$ (Table S3).

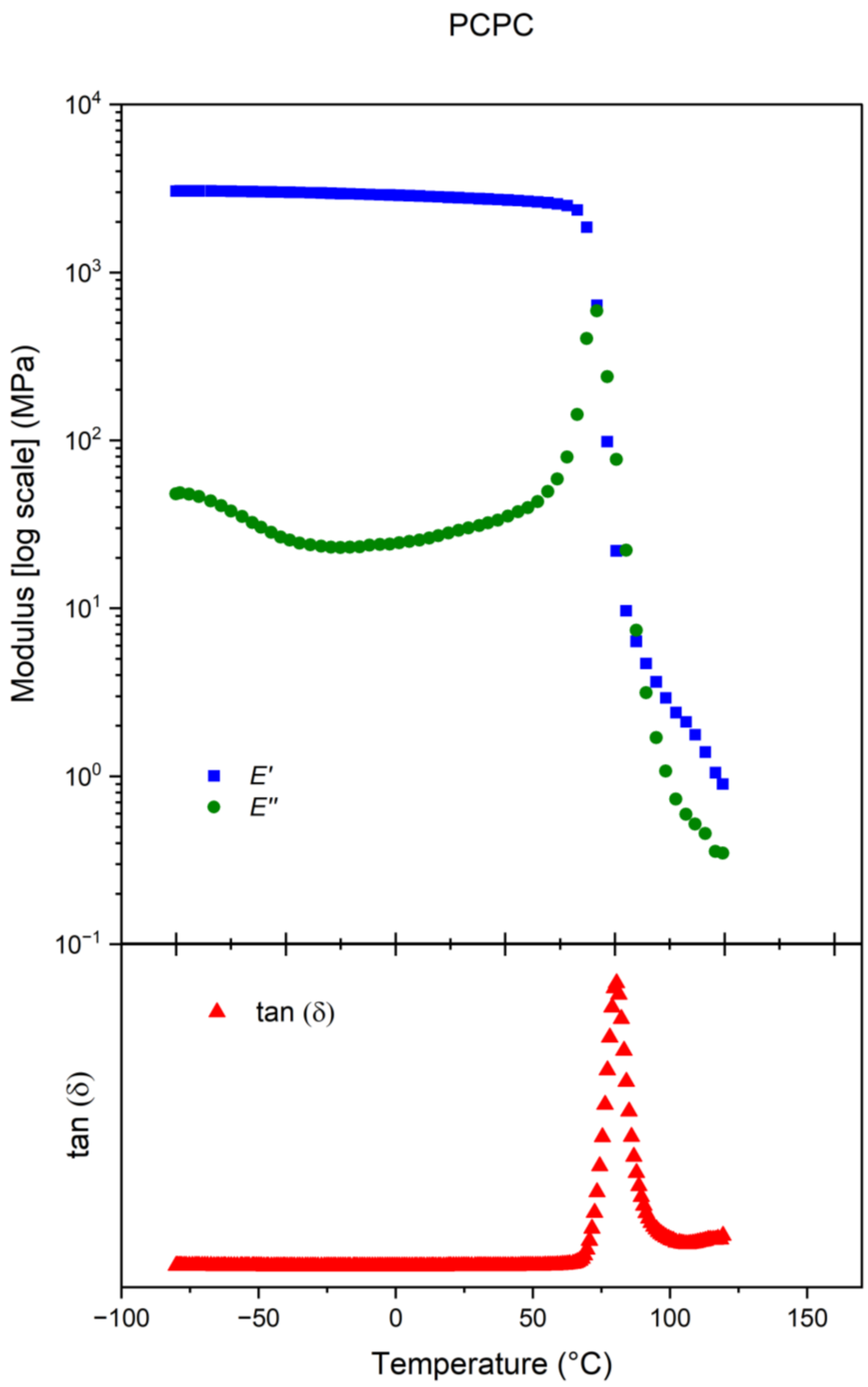

Figure S14: Dynamic mechanical temperature analysis (DMTA) temperature sweeps for PCPC (Table S3).

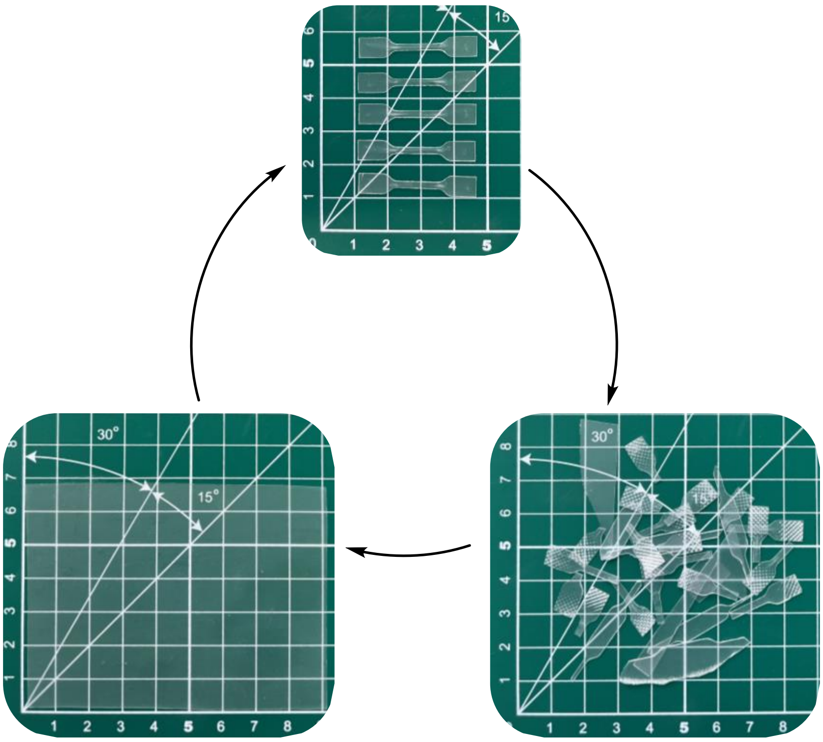

Figure S15: Photographs of $PCHC_{0.51}$-*grad*-$PCPC_{0.49}$ throughout the compression moulding and mechanical recycling process. Film thickness ~0.2 mm.

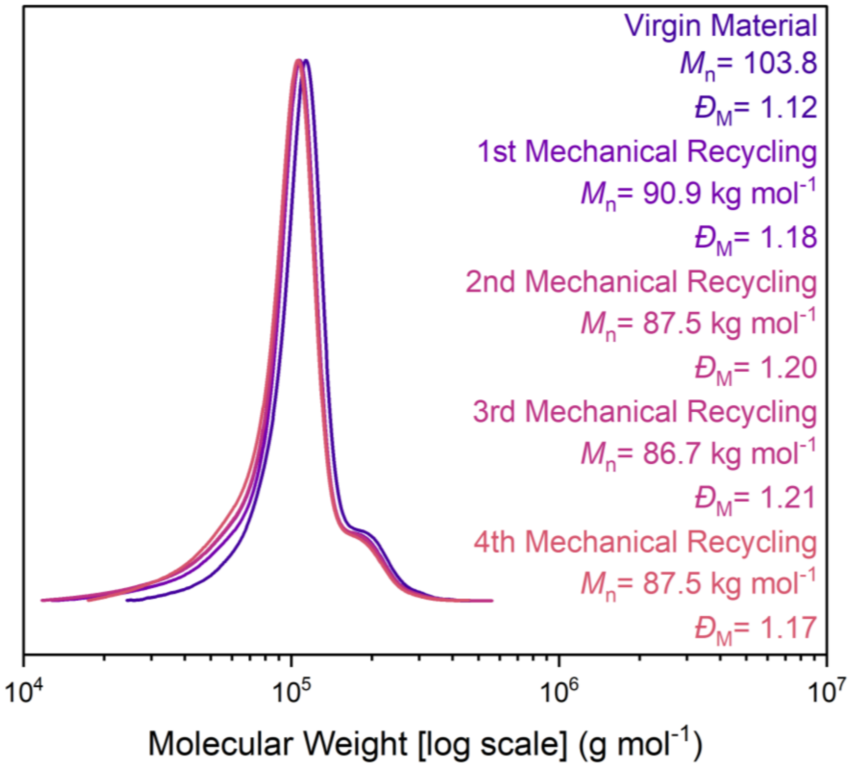

Figure S16: SEC (THF, 1 mL min^{-1}) traces for the $PCHC_{0.51}$-*grad*-$PCPC_{0.49}$ after each cycle of mechanical reprocessing. The SEC instrument is calibrated with poly(styrene) standards.

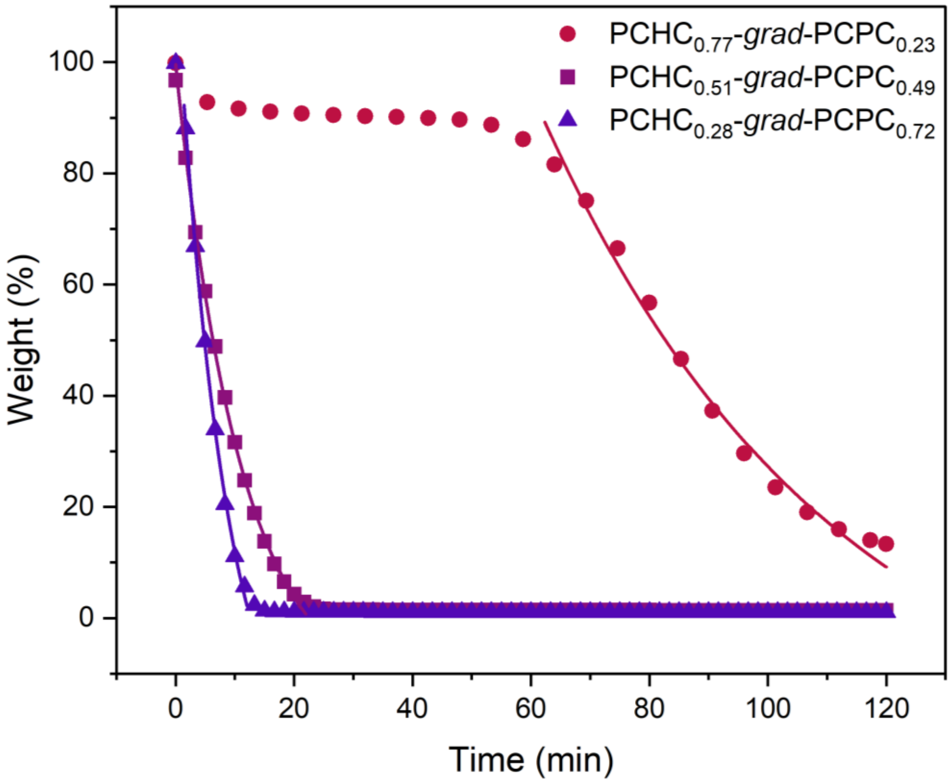

Figure S17: Solid-state depolymerization data for $PCHC_{0.51}$-*grad*-$PCPC_{0.49}$ terpolymers using [$LCoMg(OAc)_2$] catalyst (1:300), at 140 ˚C. Plots show terpolymer mass loss data vs. time. The data is fit to exponentials to determine the pseudo first order rate constants, k_{obs}.

Table S4: Data for the terpolymer depolymerizations using the Co(II)Mg(II) Catalyst

**Polymer**	**$^{a}k_{obs}$ / h^{-1}**	**^{b}TOF / h^{-1}**
$PCHC_{0.77}$-*grad*-$PCPC_{0.23}$	1.79 ± 0.19	270 ± 5
$PCHC_{0.51}$-*grad*-$PCPC_{0.49}$	5.93 ± 0.99	926 ± 14
$PCHC_{0.28}$-*grad*-$PCPC_{0.72}$	9.40 ± 1.68	1653 ± 201

$^{a}k_{obs}$ calculated from exponential fits to plots of terpolymer mass loss vs time. [b]Mass loss rate = mass of PCHC consumed (20–80% conversion)/mass of catalyst/time. Errors are reported from the mean and standard deviations of values determined from three repeat experiments.

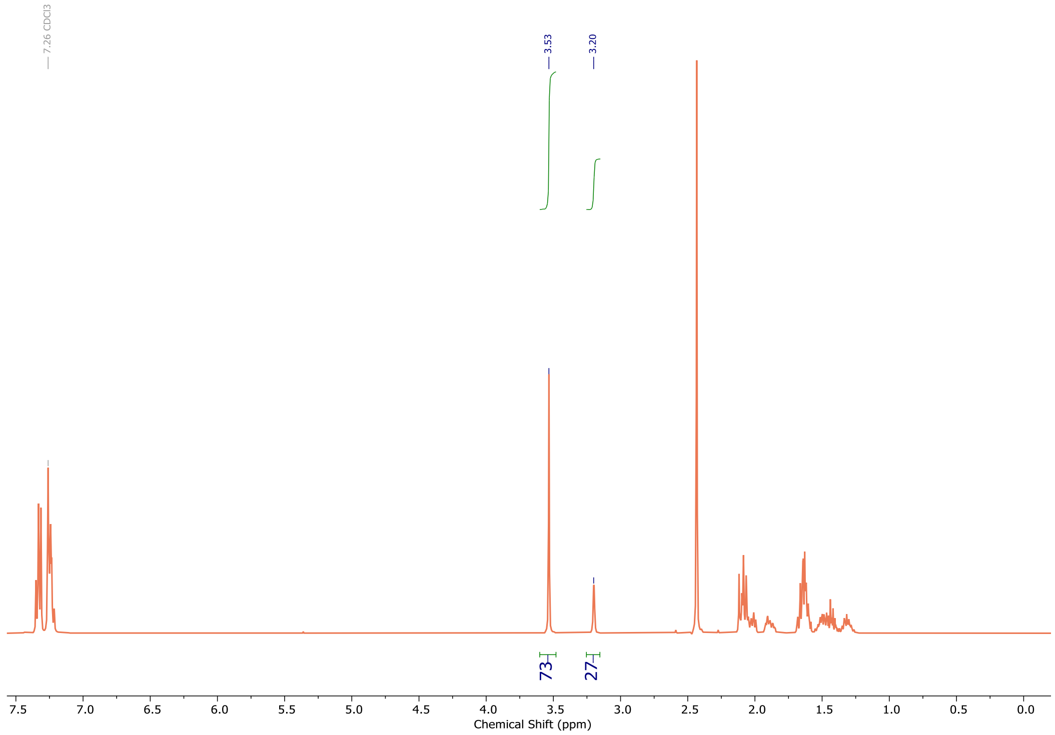

Figure S18: ^{1}H NMR spectrum (400 MHz, $CDCl_3$) of the CHO and CPO isolated from the depolymerization. The relative ratio, indicated by the integrals, is CHO:CPO = 73 : 27.

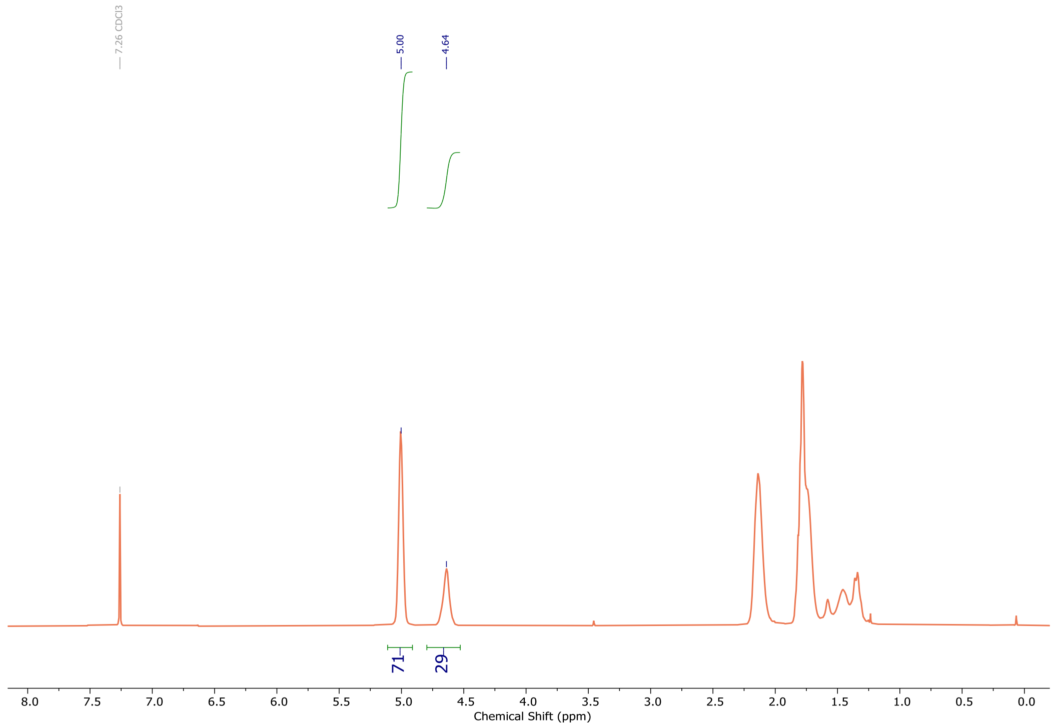

Figure S19: ^{1}H NMR spectrum (400 MHz, $CDCl_3$) of the chemically recycled (i.e. re-polymerized) PCHC-*grad*-PCPC terpolymer.

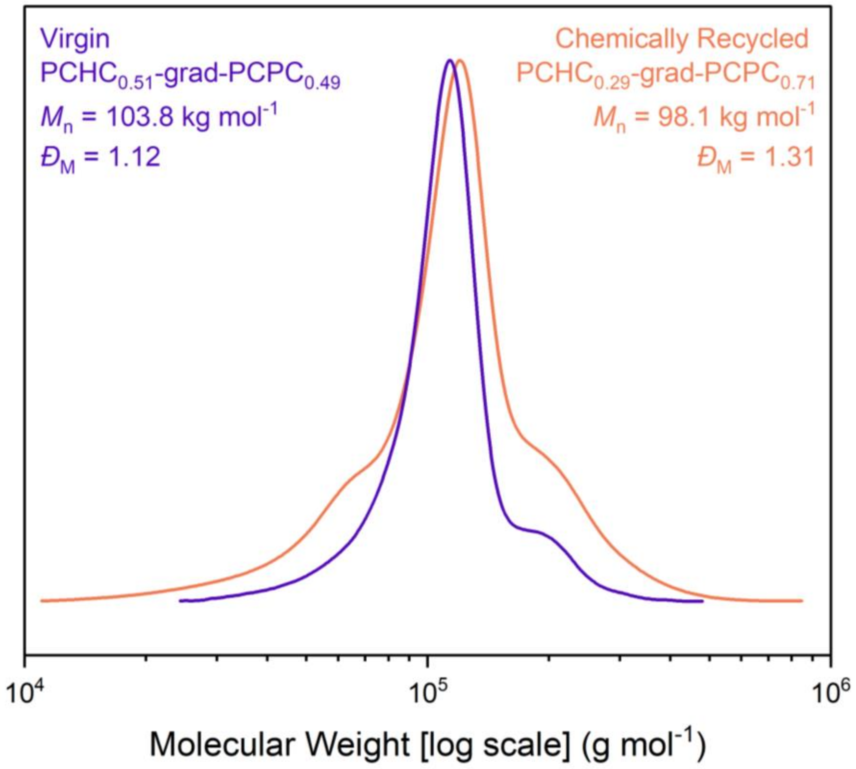

Figure S20: SEC (THF, 1 mL min^{-1}) traces for the $PCHC_{0.51}$-*grad*-$PCPC_{0.49}$ and chemically recycled $PCHC_{0.29}$-*grad*-$PCPC_{0.71}$.

The low molar mass shoulder is tentatively attributed to the initiation of trace quantities of acetic acid which result from the acetate co-ligands from the Co(II)Mg(II) depolymerization catalyst. The high molar mass shoulder, which is also seen in the SEC traces for all terpolymers, is believed to arise from residual cyclopentene diol (CPD) present in the CPO monomer. The slight difference in initiation time between CPD and CHD is responsible for the observed SEC traces. However, it is important to highlight that these protic impurities are only present at trace levels and despite this, the overall molar mass is very high (98 kg mol^{-1}), above M_c and the chemically recycled material displays equivalent mechanical properties to that of the virgin material.

Table S5: Data for the Chemically Recycled PCHC-*grad*-PCPC Terpolymers

Polymer	[a]E_Y /GPa	[b]σ /MPa	[c]ε_b /%	[d]U_T /MJ m^{-3}
$PCHC_{0.51}$-*grad*-$PCPC_{0.49}$ (Virgin Material)	1.54 ± 0.15	46.2 ± 3.1	6.5 ± 0.9	2.34 ± 0.56
1st Mechanical Recycling	1.06 ± 0.1	44.2 ± 2.4	7.1 ± 1.2	2.16 ± 0.44
2nd Mechanical Recycling	0.88 ± 0.09	45.5 ± 0.7	9.3 ± 0.5	2.95 ±0.28
3rd Mechanical Recycling	1.08 ± 0.08	45.2 ± 3.2	4.6 ± 1.4	2.48 ± 0.42
4th Mechanical Recycling	1.24 ± 0.15	44.6 ± 3.3	7.2 ± 1.2	2.63 ± 0.72
$PCHC_{0.29}$-*grad*-$PCPC_{0.71}$ (Chemically Recycled)	1.21 ± 0.04	48.8 ±7.6	8.5 ± 1.2	3.01 ± 0.58

Specimens suitable for uniaxial tensile testing were solvent cast, dried and compression moulded (150 ˚ C, 1.2 ton m^{-2}, 60 min). Measurements were conducted independently on 10 specimens and values are reported as the mean and standard deviation from those experiments. [a]Young's modulus. [b]Tensile strength. [c]Strain at break. [d]Tensile toughness (area under the stress-strain curve).

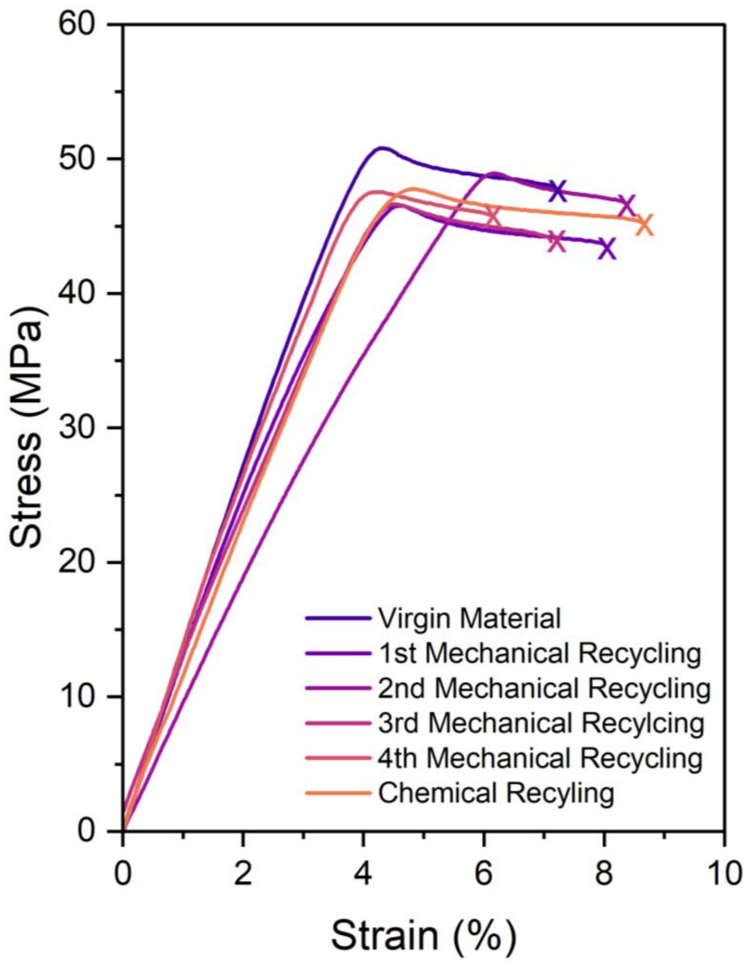

Figure S21: Representative stress-strain data (10 mL min^{-1}) for $PCHC_{0.51}$-*grad*-$PCPC_{0.49}$ after repeated cycles of mechanical reprocessing and chemical recycling.

References

(1) Spyros, A.; Argyropoulos, D. S.; Marchessault, R. H. A study of poly(hydroxyalkanoate)s by quantitative P-31 NMR spectroscopy: Molecular weight and chain cleavage. *Macromolecules* **1997**, *30* (2), 327-329. DOI: DOI 10.1021/ma9601979.

(2) McGuire, T. M.; Deacy, A. C.; Buchard, A.; Williams, C. K. Solid-State Chemical Recycling of Polycarbonates to Epoxides and Carbon Dioxide Using a Heterodinuclear Mg(II)Co(II) Catalyst. *J. Am. Chem. Soc.* **2022**, *144* (40), 18444-18449. DOI: 10.1021/jacs.2c06937.

(3) Sulley, G. S.; Gregory, G. L.; Chen, T. T. D.; Peña Carrodeguas, L.; Trott, G.; Santmarti, A.; Lee, K. Y.; Terrill, N. J.; Williams, C. K. Switchable Catalysis Improves the Properties of CO2-Derived Polymers: Poly(cyclohexene carbonate- b-ϵ-decalactone- b-cyclohexene carbonate) Adhesives, Elastomers, and Toughened Plastics. *J. Am. Chem. Soc.* **2020**, *142* (9), 4367-4378. DOI: 10.1021/jacs.9b13106.

(4) Deacy, A. C.; Kilpatrick, A. F. R.; Regoutz, A.; Williams, C. K. Understanding metal synergy in heterodinuclear catalysts for the terpolymerization of CO2 and epoxides. *Nat. Chem.* **2020**, *12* (4), 372-380. DOI: 10.1038/s41557-020-0450-3.

(5) Fetters, L. J.; Lohse, D. J.; Richter, D.; Witten, T. A.; Zirkel, A. Connection between Polymer Molecular-Weight, Density, Chain Dimensions, and Melt Viscoelastic Properties. *Macromolecules* **1994**, *27* (17), 4639-4647. DOI: DOI 10.1021/ma00095a001.

(6) *Modern Plastics: Encyclopedia' 96 with Buyers' Guide*; McGraw-Hill, 1995.

(7) Koning, C.; Wildeson, J.; Parton, R.; Plum, B.; Steeman, P.; Darensbourg, D. J. Synthesis and physical characterization of poly(cyclohexane carbonate), synthesized from CO_2 and cyclohexene oxide. *Polymer* **2001**, *42* (9), 3995-4004. DOI: 10.1016/S0032-3861(00)00709-6.
